# Extensive migration of young neurons into the infant human frontal lobe

**DOI:** 10.1126/science.aaf7073

**Published:** 2016-10-07

**Authors:** Mercedes F. Paredes, David James, Sara Gil-Perotin, Hosung Kim, Jennifer A. Cotter, Carissa Ng, Kadellyn Sandoval, David H. Rowitch, Duan Xu, Patrick S. McQuillen, Jose-Manuel Garcia-Verdugo4, Eric J. Huang, Arturo Alvarez-Buylla

**Affiliations:** 1 Edythe Broad Institute for Stem Cell Research and Regeneration Medicine University of California San Francisco CA 94143 USA; 2 Department of Neurology, University of California San Francisco CA 94143 USA; 3 Department of Neurological Surgery University of California San Francisco CA 94143 USA; 4 Laboratory of Comparative Neurobiology Instituto Cavanilles, Universidad de Valencia CIBERNED Valencia Spain; 5 Multiple Sclerosis and Neural Regeneration Unit Department of Neurology Hospital Universitario y Politecnico La Fe 46026 Valencia Spain; 6 Department of Radiology and Biomedical Imaging University of California San Francisco CA 94143 USA; 7 Department of Pathology University of California San Francisco CA 94143 USA; 8 Department of Pediatrics University of California, San Francisco CA 94143 USA; 9 Department of Paediatrics University of Cambridge Cambridge CB2 0QQ, UK

## Abstract

The first few months after birth, when a child begins to interact with the environment, are critical to human brain development. The human frontal lobe is important for social behavior and executive function; it has increased in size and complexity relative to other species, but the processes that have contributed to this expansion are unknown. Our studies of postmortem infant human brains revealed a collection of neurons that migrate and integrate widely into the frontal lobe during infancy. Chains of young neurons move tangentially close to the walls of the lateral ventricles and along blood vessels. These cells then individually disperse long distances to reach cortical tissue, where they differentiate and contribute to inhibitory circuits. Late-arriving interneurons could contribute to developmental plasticity, and the disruption of their postnatal migration or differentiation may underlie neurodevelopmental disorders.

Local inhibitory interneurons in the cerebral cortex play key roles in the final assembly of brain circuits, and their maturation is essential to critical-period plasticity and learning (1, 2). Interneurons are born in ventral progenitor zones, primarily the medial and caudal ganglionic eminences (MGE and CGE), and then migrate dorsally to reach the cerebral cortex (3–7). Neuronal migration is largely completed during fetal development (8, 9). However, in many species, migrating young neurons persist in the postnatal subventricular zone (SVZ) of the lateral ventricles (10, 11). In rodents, SVZ-derived neurons migrate along the rostral migratory stream (RMS) into the olfactory bulb, where they replace neurons throughout life (12–15). A small number of these neurons, born perinatally, migrate into the anterior forebrain to become small axonless neurons (16,17) or into the ventral forebrain to become granule cells in the islands of Calleja (18). In the infant human brain, SVZ-derived young neurons migrate along the RMS (19, 20) into the olfactory bulb, and a sub-population of these cells migrates along a medial migratory stream (MMS) into the ventral medial prefrontal cortex (20). The postnatal human SVZ extends dorsally, but it is not known whether cells in this region also contribute to other areas of the human forebrain. Given the tremendous postnatal growth of the human frontal lobe and the prevalence of migrating young neurons in the adjacent SVZ, we investigated whether neurons also continue migrating into the frontal lobe of infants and young children.

## Postnatal migratory pathways into the frontal lobes

**Fig. 1:**
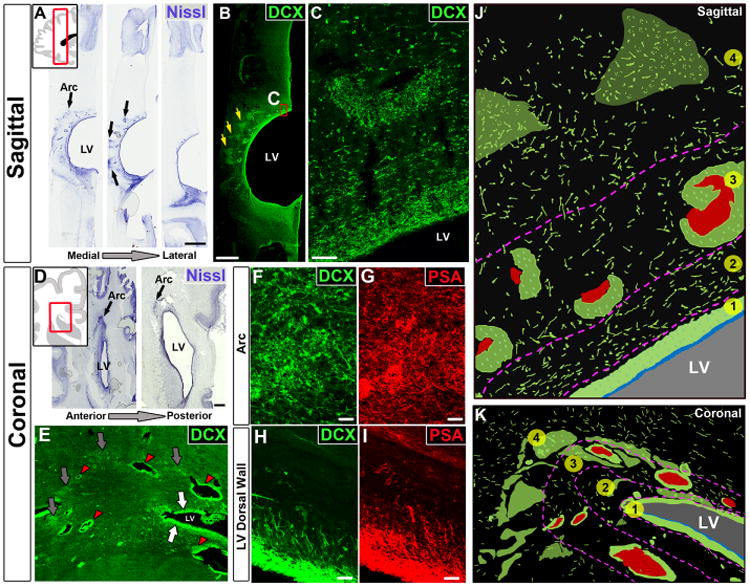
Mi**grating young neurons in the infant frontal lobe are widely distributed in four tiers.** (**A**) Serial Nissl-stained sections (taken at birth) reveal cell-dense collections around the anterior body of the lateral ventricle (black arrows, defined here as the Arc); LV, lateral ventricle. (**B** and **C**) The cells in these densities (yellow arrows) and next to the ventricular wall express DCX. (**D**) Coronal sections (38 GW) showing cell densities close to the ventricular wall (eyebrow-shaped, black arrows). (**E**) Dense aggregates of DCX^+^ cells around the walls of the lateral ventricles (white arrows), around blood vessels (red arrowhead), and in the parenchyma within the Arc (gray arrows). (**F** to **I**) DCX^+^ cells also express PSA-NCAM; (F) and (G) show cells within the Arc; (H) and (I) show cells next to the ventricular walls. (**J** and **K**) Schematic drawings of traced DCX^+^ cells (in green) illustrating how cells within the Arc are organized into four tiers (see text). Blood vessels are shown in red; light green clusters correspond to DCX^+^ cellular densities seen in (B) and (E). Scale bars, 2 mm [(A) and (B)], 50 μm (C), 1 mm (D), 25 μm [(F) to (I)].

In samples from the anterior forebrain of children younger than 3 months of age, regions of high cell densities were observed in the SVZ. These densities were adjacent to the anterior body of the lateral ventricle and within the neighboring subcortical white matter, forming a distinct arching structure in sagittal sections or an eyebrow-shaped extension in coronal sections (Fig. 1, A and D, black arrows). The majority of cells within these regions coexpressed double-cortin (DCX) and polysialylated neural cell adhesion molecule (PSA-NCAM), markers of young migrating neurons (Fig. 1, B, C, and E, and fig. S1B) (21, 22). Many of these cells displayed migratory morphology, with an elongated cell body and a leading process that was occasionally bifurcated (23–25). DCX^+^ cells did not express Olig2 (see below), which marks oligodendrocytes and their precursor cells, nor the astrocytic markers glial fibrillary acidic protein (GFAP) and Aldh1L1 (fig. S1 and fig. S2,K and L).

**Fig. 2:**
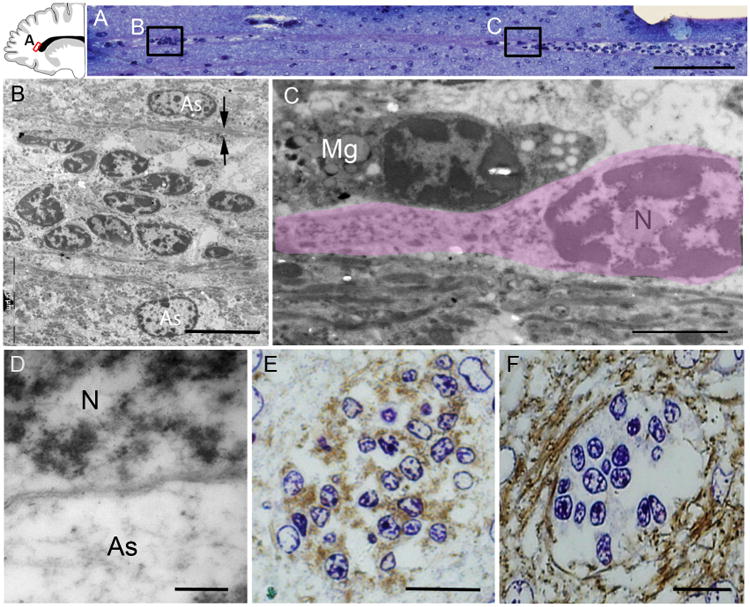
Arc cells have ultra-structural features of migrating young neurons (**A**) Toluidine blue staining of a semithin sagittal section from a 1-month-old brain showing a chain of cells around a blood vessel in tier 3 (see Fig. 1). Locations of images in (B) and (C) are shown. (**B**) Electron microscopy shows that this chain is made up of elongated cells with ultrastructural features of young migrating neurons; the chain is flanked by astrocytes (As) whose expansions (arrows) contain intermediate filaments. (**C**) An elongated migrating neuron (outlined in pink) next to a microglial cell (Mg). Migrating young neurons (N) frequently had an elongated morphology, a leading process, poly-ribosomes, and no intermediate filaments. (**D**) The cytoplasm of astrocytes is lighter and contains intermediate filaments. (**E** and **F**) 3,3′-Diaminobenzidine (DAB) staining of semithin coronal sections (adjacent to those used for electron microscopy) shows DCX expression within the chain and GFAP expression surrounding them; the counterstain is toluidine blue. Scale bars, 50 μm (A), 10 μm (B), 2 μm (C), 200 nm (D), 15 μm [(E) and (F)].

In postmortem brains collected at birth and at 1 month, these putative migrating young neurons were organized into four layers, or tiers, around the anterior body of the lateral ventricles (Fig. 1, J and K, and fig. S1F). Tier 1 corresponded to a cell-dense SVZ band of DCX^+^ cells next to the walls of the lateral ventricle; between 6 and 12 months, tier 1 is depleted of young neurons, becoming a hypocellular gap layer (20). Tier 2 contained a more dispersed collection of DCX^+^ cells. Tier 3 was an intermediate region with many DCX^+^ cells within clusters, frequently around blood vessels, and dispersed DCX^+^ cells around these clusters (fig. S3). Tier 4 contained a group of DCX^+^ cells dispersed within areas of the developing white matter. Many cells in tier 4 were organized around radial finger-like extensions of triangular shape (Fig. 1B, yellow arrows). We analyzed these tiered regions in 1-day-old and 28-day-old brains by electron microscopy. Cells with the ultrastructure of young migrating neurons were found throughout tiers 1 to 4. Migrating young neurons were organized as chains (12) or as individual cells (Fig. 2, A to D, and fig. S4, C and G). Those within chains had adherent junctions similar to those observed in the RMS (fig. S4, G and H). Confocal and electron microscopy showed that chains of migrating neurons were flanked by cells rich in intermediate filaments containing GFAP (Fig. 2F, fig. S1C, and movie S1).

To generate a multiplanar representation of migratory streams of cells, we used high-resolution magnetic resonance imaging (MRI) to image intact hemispheres from postmortem human brains between birth and 2 months of age, including a premature case born at 34 gestational weeks (GW) (table S1). MRI analysis revealed a T2 hyperintense signal adjacent to the anterior horn of the lateral ventricle (fig. S5, A and B, red shading). Three-dimensional rendering of the segmented areas of T2 signal in brains at 34 GW and at birth showed that this structure formed a cap around the anterior horn of the lateral ventricle (fig. S5D). In sagittal MRI planes, this cap structure had an arc shape (fig. S5, A and G), running parallel to the anterior cingulate cortex and extending caudally to approximately the level of the central sulcus. This arc was also observed in live MRI images of the developing human brain (fig. S5, H and I). The T2 hyper-intense signal was localized to ventricular regions densely populated by DCX^+^ cells (fig. S1, E to G). Given the organization we observed in both histological and radiographic images, we refer to these streams of cells as the “Arc.”

## Migratory features of young neurons in the human infant brain

**Fig. 3:**
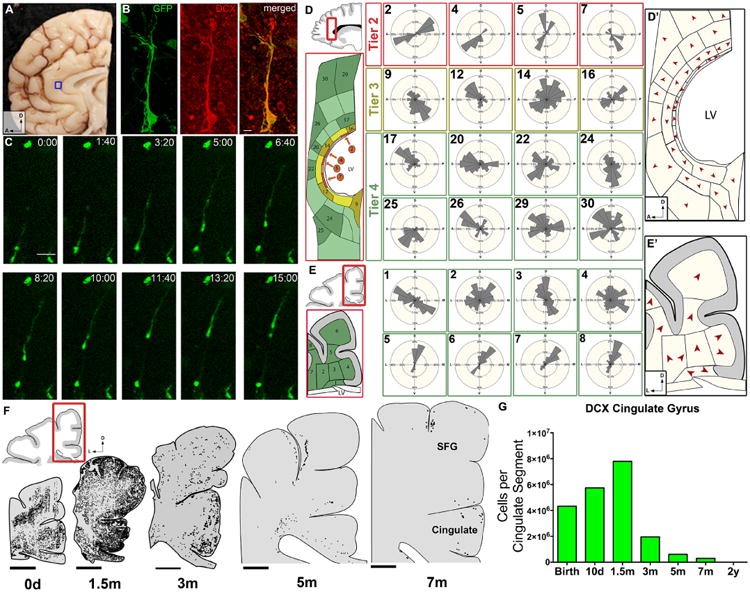
Migration and directionality of young neurons in the infant brain (**A**) Boxed region shows area of the neonatal brain that was imaged in (B) and (C) in the cingulate gyrus. (**B**) DCX^+^ adenoGFP-labeled cell with migratory morphology. (**C**) Time-lapse sequence (15 hours) of adenoGFP-labeled cell revealing leading process extension, nucleokinesis, and trailing process retraction. This cell traveled ∼100 μm, migrating anteriorly in the sagittal plane. (**D** and **E**) Vector mapping of orientation of DCX^+^ cell leading processes, in sagittal and coronal sections; note how directionality changes in the different tiers. See figs. S6 and S7 for complete analysis. (**D**′ and **E**′) Red arrowheads indicate the modal (most frequent) direction of DCX^+^ cells' leading process. (**F**) Spatiotemporal mapping of DCX^+^ cells in coronal cortical sections; between birth and 1.5 months, many DCX^+^ cells have moved from the periventricular and parenchymal regions into the developing cortex of the cingulate and superior frontal gyrus. DCX^+^ cells then rapidly decrease at 3 and 5 months, but a few DCX^+^ cells with clear migratory morphology remain at 7 months. (**G**) Quantification of DCX^+^ cells in the cingulate gyrus (white matter and gray matter). Scale bars, 10 μm (B), 50 μm (C), 5 mm (F). Directional axes: D, dorsal; L, lateral; A, anterior.

To confirm that these cells were in fact actively migrating, we obtained human neonatal brain samples (table S1) with short postmortem intervals and infected them with adenovirus carrying green fluorescent protein (adenoGFP) for time-lapse confocal microscopy. Elongated GFP^+^ cells (*n* = 18) with leading processes were identified, and we studied their behavior for 24 to 48 hours (Fig. 3C). As shown (movies S2 and S3), these cells actively migrated in coronal and sagittal slice cultures, displaying leading process extension, nucleokinesis, and retraction of trailing process. These features were indistinguishable from the migratory behavior of neurons in the fetal brain (24, 26, 27). Active migration was also observed within clusters of cells (movies S3 and S4) at the dorsolateral ventricular edge, but because of their high cellular density, the behavior of individual cells was often not evident. In one of these clusters, we captured a labeled cell escaping the cluster to begin individual migration (movie S4). Immunostaining of these brain slices after time-lapse imaging confirmed that the migrating cells were DCX^+^ (Fig. 3B). Thus, neurons in the newborn brain within the Arc and immediate surroundings are actively migrating.

Using fixed tissue, we inferred possible migratory trajectories from the orientation of the leading process of DCX^+^ young neurons. We defined a vector from the center of the cell body in the direction of the leading process (see supplementary materials). We applied this analysis to DCX^+^ cells in coronal and sagittal sections at birth and 1.5 months of age in periventricular and subcortical white matter regions in the frontal lobe (Fig. 3, D and E). We observed that the vector orientation of the cells changed depending on the region. The leading process of DCX^+^ cells could not be discerned in tier 1 because of the high cellular density, but the majority of cells in tier 2 appeared to be migrating tangentially, parallel to the ventricle wall. In the sagittal plane, cells were oriented ventrally and dorsally. In tier 3, the orientation remained largely tangential, but cellular direction was more variable than in tier 2. Lastly, in tier 4 and at the gray matter–white matter junction, more cells were oriented toward the developing cortex (Fig. 3, D and E, and figs. S6 and S7). A similar pattern of vector orientation was also observed in the coronal plane of the frontal lobe at 1.5 months (fig. S8). These data suggest that young neurons in regions close to the ventricles primarily migrate in the tangential plane, whereas those in tiers 3 and 4, and in the developing white matter and cortex, are more widespread and cortically directed.

We next mapped the distribution of young migratory neurons adjacent to the ventricular wall and in the overlying cortices at birth and at 1.5, 3, 5, and 7 months. At and immediately after birth, elongated DCX^+^ cells were found at the dorsal ventricular wall and in the mantle region of the developing white matter (Fig. 3F). By 1.5 months, DCX^+^ cells were mainly found in the dorsal cortex in the superior and middle frontal gyri and the cingulate cortex, but many remained in the developing white matter. The total number of DCX^+^ cells with migratory morphology decreased between 1.5 and 7 months of age (Fig. 3, F and G; for representative DCX^+^ cells at 5 and 7 months, see fig. S9). The entry of DCX^+^ cells into the anterior cingulate gyrus was correlated with an increase in the number of cells expressing NeuN, a marker of mature neurons (fig. S10B). We also examined the cingulate cortex at 2, 6, and 15 years of age. Four to six DCX^+^ cells were observed per section in the 2-year-old sample, but these cells did not have a clear migratory morphology. None were detected at 6 or 15 years. Sagittal sections mapped at birth also demonstrated migrating young neurons moving into the anterior pole of the developing human brain (fig. S11). These observations indicate that postnatal neuronal migration in the human frontal lobe, in the Arc and beyond, occurs primarily within the first 3 months after birth, with a few DCX^+^ elongated cells persisting at 7 months.

## Postnatally migrating neurons differentiate into interneurons

**Fig. 4:**
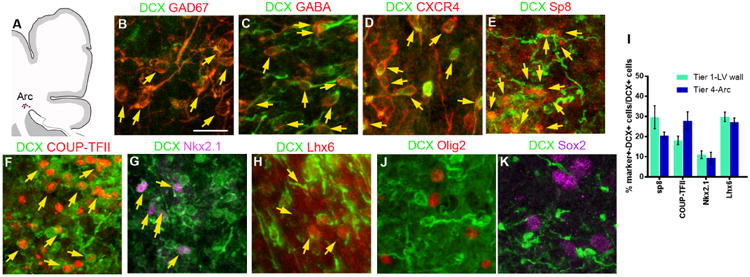
Interneuron and subpallial marker expression in migrating DCX+ cells in the infant brain (**A**) Schematic of coronal section indicating the Arc area that was analyzed at the dorsolateral edge of the ventricle; see fig. S2 for marker expression next to the walls of the lateral ventricle. (**B** to **D**) DCX^+^ cells express GAD67, GABA, and the cytokine receptor CXCR4 present in migrating interneurons. (**E** to **H**) Subpopulations of DCX^+^ cells express different transcription factors associated with ventral telencephalic origin, including Sp8, COUP-TFII, Nkx2.1, or Lhx6 associated with the CGE or MGE. (**I**) Quantification of DCX^+^ cells expressing Sp8, COUP-TFII, Nkx2.1, and Lhx6. Bars show means ± SEM of counts performed on three or four individual cases. (**J** and **K**) DCX^+^ cells do not express Olig2 or Sox2. Scale bar, 20 μm.

We sought to determine which types of neurons the DCX^+^ cells in the Arc become. DCX^+^ cells in all tiers at birth and at 1.5 months expressed γ-aminobutyric acid (GABA), the main inhibitory neurotransmitter in the adult brain; GAD67, an enzyme involved in the production of GABA; and the chemokine receptor CXCR4, seen in migrating interneurons (Fig. 4, B to D). Within tiers 1 and 2 (close to the ventricular wall), 92.5 ± 2.9% (SD) of DCX^+^ cells were GAD67^+^ and 96.1 ± 2.4% were GABA^+^. Farther away, within tiers 3 and 4, 91.2 ± 4.4% of DCX^+^ cells were GAD67+ and 94.8 ± 5.8% were GABA^+^. Because cortical interneurons primarily arise from the MGE and CGE (3, 5,19, 22), we asked whether DCX^+^ cells in the Arc expressed Nkx2.1 or Lhx6 (transcription factors associated with the MGE), or Sp8 and COUP-TFII [associated with the CGE and possibly the lateral ganglionic eminence (LGE)]. At birth, about 10% of DCX^+^ cells were Nkx2.1^+^ and 28% were Lhx6^+^ (Fig. 4, G to I, and fig. S2, F and G). Sp8 and COUP-TFII were expressed in 24% and 22% of DCX^+^ cells, respectively (Fig. 4, E, F, and I, and fig. S2, D and E). DCX^+^ cells did not express Sox2 or Tbr2, transcription factors associated with early and intermediate progenitor cells, respectively (Fig. 4K and fig. S2), nor did they express Emx1, CTIP2, or SATB2, transcription factors associated with excitatory neurons (fig. S2). In tiers 1 to 4 at birth, we found very few cells positive for Ki67, a marker of proliferating cells (fig. S12). Most of these Ki67+ cells were also Olig2^+^ and none were DCX^+^. Thus, DCX^+^ cells in the postnatal frontal lobe correspond to postmitotic migrating young inhibitory interneurons, likely derived from the developing ganglionic eminences (CGE, MGE, and possibly LGE).

## The interneuron subtype composition in the anterior cingulate cortex changes postnatally

**Fig. 5:**
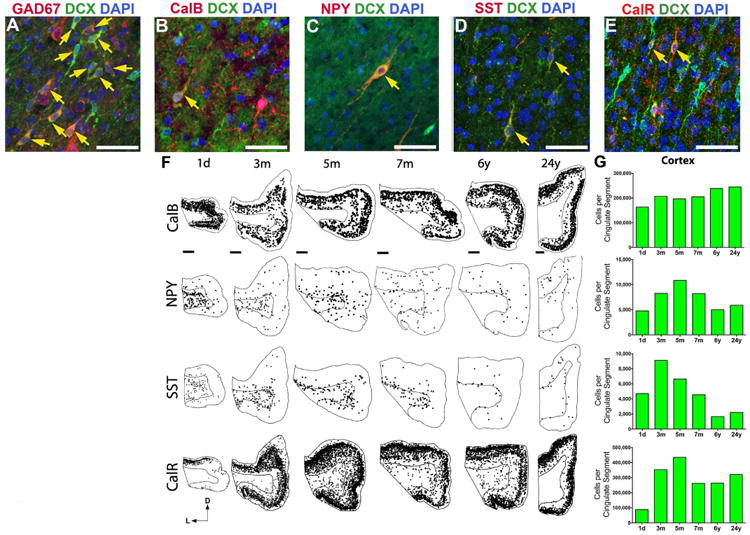
Interneuron subtype development in the cingulate gyrus (**A** to **E**) Many DCX^+^ cells in the neonatal cingulate cortex express GAD67 (A), and sub-populations also coexpress interneuron subtype markers: calbindin (CalB) (B), neuropeptide Y (NPY) (C), somatostatin (SST) (D), and calretinin (CalR) (E). DAPI, 4′,6-diamidino-2-phenylindole. Yellow arrows point to DCX^+^ cells that coexpress the indicated subtype markers. (**F**) Spatiotemporal distribution of interneuron subtypes from birth to 24 years. NPY^+^ and SST^+^ cells are located primarily in the white matter at birth but shift to the cortex over time. CalR^+^ and CalB^+^ are already expressed in cells throughout the cortex at all ages, but their number continues to increase during the first five postnatal months. (**G**) Stereological quantification of interneuron subtypes in the cingulate cortex from birth to 24 years. The number of NPY^+^, SST^+^, CalB^+^, and CalR^+^ cells increases between birth and 5 months, coinciding with the arrival of DCX^+^ cells in the cingulate cortex (see Fig. 3G). Scale bars, 50 μm [(A) to (E)], 2 mm [(F), 1 day to 6 years], 1 mm [(F), 24 years]. Directional axes: D, dorsal; L, lateral.

To address how the Arc might contribute to developing cortical circuits, we mapped and quantified the total number of cells, neurons, and interneuron subtypes from birth until adulthood. We focused on the anterior cingulate cortex, which runs parallel to the Arc and had many DCX^+^ cells during the first postnatal months. The cell number and volume of the cingulate cortex increased between birth and 5 months of age (fig. S10, A and C). The neuronal population in the cingulate cortex, as identified by NeuN expression, also increased during this time. These population changes followed the peak in the total number of DCX^+^ cells, at ∼1.5 months, suggesting that the cingulate cortex receives young migratory neurons up to 5 months after birth. Most DCX^+^ cells found postnatally in the cingulate cortex white matter expressed GAD67, and a subpopulation expressed interneuron subtype markers [neuropeptide Y (NPY), somatostatin (SST), calretinin (CalR), or calbindin (CalB)] (Fig. 5, A to E). If these different subtypes of migrating young neurons enter the cingulate cortex, we hypothesized that its interneuron subtype composition would change over time. Indeed, by quantifying the abundance of different interneuron subtypes in this region, we found that the number of cells expressing NPY, SST, CalR, and CalB increased during the first 5 months after birth (Fig. 5, F and G). The number of parvalbumin-expressing cells also changed with age (from ∼20,000 cells per cingulate segment at 3 months to >72,000 cells at 24 years), but we do not know whether this increase is due to cell addition or due to their late maturation (28,29). These data suggest that DCX^+^ cells from the Arc contribute to interneuron subtype populations within the infant cingulate cortex.

## Discussion

**Fig. 6:**
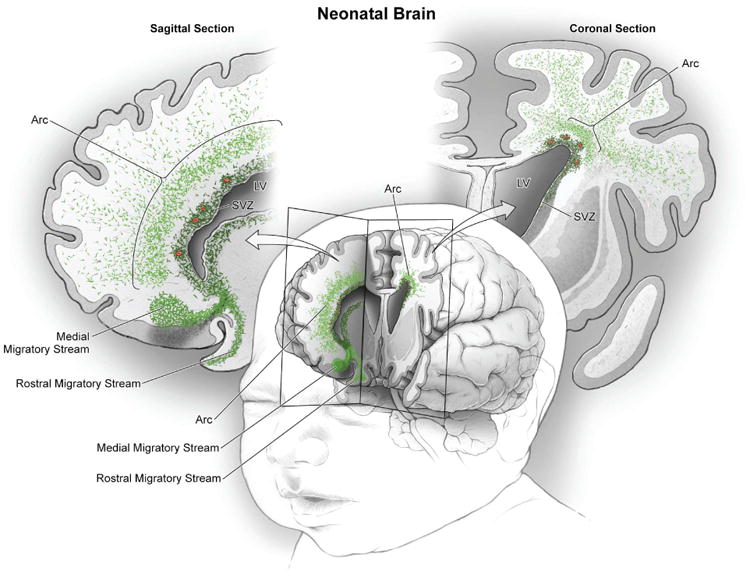
Migratory streams of young neurons in the frontal lobe of the early postnatal human brain In the frontal lobe of the neonatal human brain, cut in sagittal and coronal planes in this schematic, large numbers of young migrating neurons persist (shown in green) (see Figs. 1 to 3). Multiple concentric tiers of migrating cells are observed around the anterior pole of the lateral ventricle (see Fig. 1). Close to the ventricular wall, migrating young neurons are largely oriented tangentially; dense subpopulations are also clustered around blood vessels (red). Farther out, young neurons are more dispersed, many now oriented radially; they appear to migrate long distances through the developing white matter to reach the cortex. Ventrally, we also illustrate the RMS and the MMS, which target the olfactory bulb and medial prefrontal cortex, respectively (20).

We have identified a large, heterogeneous population of late-migrating neurons in the infant human brain that targets an extensive region of the anterior forebrain, including the cingulate gyrus and prefrontal cortex. In the rodent cortex, a population of CGE-derived young migrating neurons continues to migrate into the cortex within the first few weeks of postnatal life (16,17, 30). The population of young migrating neurons in the frontal lobe of postnatal humans appears to include this population but also others, including SST, NPY, and CalB. This assortment of subtypes, along with the expression of the regionally specific transcription factors Nkx2.1, Lhx6, COUP-TFII, and SP8, suggests that cells within the Arc derive from various progenitor zones in the ventral forebrain. The extensive tangential migration in the SVZ and perivascular region of the infant brain (Fig. 6 and movie S5) could allow for mixed populations of interneurons from distinct progenitor zones (31) to reach appropriate cortical regions. The precise time and birthplace of young migrating neurons within the postnatal human frontal lobe remains to be determined.

Because migrating neurons from the Arc reach cortical circuits during postnatal life, sensory experience could shape their recruitment and possibly their connectivity (32–36). Periods of plasticity are tightly linked to the time course of inhibitory interneuron maturation; thus, the late incorporation of inhibitory neurons into the frontal cortex could also be associated with the extension and delay in periods of plasticity during postnatal human development (37–39). Given the large numbers of young neurons that continue to migrate in the human brain at birth and during the first few months of life, injuries during this time (e.g., hypoxic ischemia) could affect neuronal recruitment from the Arc (40, 41) and may contribute to sensorimotor handicaps and neurocognitive deficits, including those seen in epilepsy, cerebral palsy, and autism spectrum disorders (42, 43).

## Supplementary Material

Supplemental

Supplemental movie 1

Supplemental movie 2

Supplemental movie 3

Supplemental movie 4
