# Supplementary material for: Extensive migration of young neurons into the infant human frontal lobe
Source: Science. Author manuscript; Available in PMC 2017 May 18. (PMC5436574; doi:10.1126/science.aaf7073)
Supplement: Supplemental [file NIHMS856057-supplement-Supplemental.pdf]

www.sciencemag.org/content/354/6308/aaf7073/suppl/DC1

Supplementary Materials for

Extensive migration of young neurons into the infant human frontal lobe

Mercedes F. Paredes, David James, Sara Gil-Perotin, Hosung Kim, Jennifer A. Cotter, Carissa Ng, Kadellyn Sandoval, David H. Rowitch, Duan Xu, Patrick S. McQuillen, Jose-Manuel Garcia-Verdugo, Eric J. Huang,* Arturo Alvarez-Buylla*

*Corresponding author. Email: alvarezbuyllaa@ucsf.edu (A.A.-B.); eric.huang2@ucsf.edu (E.J.H.)

Published 7 October 2016, *Science* **354**, aaf7073 (2016)
DOI: 10.1126/science.aaf7073

This PDF file includes:

Materials and Methods

Figs. S1 to S12

Tables S1 to S4

Captions for movies S1 to S5

References

Other supplementary material for this manuscript includes:

Movies S1 to S5

Materials and Methods

Human tissue collection. Fifty de-identified human specimens were collected during autopsy, with post-mortem interval of less than 48 hours (Table S1). Tissue was collected with previous patient consent in strict observance of the legal and institutional ethical regulations of the University of California San Francisco Committee on Human Research. Protocols were approved by the Human Gamete, Embryo and Stem Cell Research Committee (institutional review board) at the University of California, San Francisco. Age at the time of tissue collection is stated in Table S1. For infant cases, when the brain is at full term (37 to 40 gestational weeks) and autopsy performed within 2 days after birth, we refer to this as "birth." Brains were cut into ~1.5 cm coronal or sagittal blocks, fixed in 4% paraformaldehyde for 2 days, and then cryoprotected in a 30% sucrose solution. Blocks were cut into 30-micron sections on a cryostat and mounted on glass slides for immunohistochemistry.

Immunohistochemistry. Frozen slides were allowed to equilibrate to room temperature for 3 hours. Some antigens required antigen retrieval (see Table S2), which was conducted at 95°C in 10 mM Na Citrate buffer, pH=6.0. Duration of antigen retrieval for each antigen can be found in Table S2. Following antigen retrieval, slides were washed with TNT (0.05% TX100 in PBS) for 10 minutes, placed in 1% H2O2 in PBS for 45 minutes, and then blocked with TNB solution (0.1 M Tris-HCl, pH 7.5, 0.15 M NaCl, 0.5% blocking reagent from PerkinElmer) for 1 hour. Slides were incubated in primary antibodies overnight at 4°C (see Table S2) and in biotinylated secondary antibodies (Jackson Immunoresearch Laboratories) for 2.5 hours at room temperature. All antibodies were diluted in TNB solution from PerkinElmer. Sections were then incubated for 30 min in streptavidin-horseradish peroxidase that was diluted (1:200) with TNB. Tyramide

signal amplification (Perkin-Elmer) was used for some antigens. Sections were incubated in tyramide-conjugated fluorophores for 5 minutes at the following dilutions: Fluorescein, 1:50; Cy3, 1:100; Cy5, 1:100.

Epifluorescence microscopy. For DCX spatiotemporal maps and interneuron subtype quantifications, entire sections were imaged at 10x (0.30 NA) on a Zeiss Axiovert 200M microscope. Image collection and analysis was done in Neurolucida software (MBF Bioscience).

Directionality maps of young migrating neurons. Individual DCX+ cells with migratory morphology were identified on fluorescent images of coronal and sagittal sections. For each DCX+ cell, we drew a vector between the soma and the end of the leading process in the direction of the cell's leading process, using Neurolucida software (MBF Bioscience). This was only done for cells in which the soma and leading process was clearly observed. We divided sections analyzed into Tiers, corresponding to anatomical subregions (e.g., subventricular, perivascular which is near blood vessels); this compartmentalization matched the gross distribution patterns of the DCX+ cells (see text, Figs. 1 and 3). Oriana software (Kovach) was used to generate compass histograms that summarize the directionality of all DCX+ cells analyzed within each region. The radial length of each wedge in a compass histogram indicates the percentage of cells oriented in a given direction.

Cell quantifications. Brains used for quantification were manually cut into coronal blocks, beginning at the mammillary body and then extending rostrally by approximately 1 cm. We refer

to the portion of the cingulate (a subregion of Brodmann Area 24) contained in these tissue blocks as a "cingulate segment" (fig. S9A, blue shaded region). From each brain, one cingulate segment was cut on a cryostat into 30-micron sections that were used for quantifications. Since our specimens were cut into tissue blocks manually by hand, these tissue blocks were often variable in thickness and yielded different numbers of sections for analysis. To account for this variability and facilitate comparisons of cell numbers across specimens, we normalized our cell counts (obtained either through exhaustive counting or the Optical Fractionator, as described below) to a standardized segment arbitrarily defined as 150 sections or 4.5 mm. (equation 1).

$$(1)\ \text{Normalized Cell Count} = \frac{(\text{Cell Count})(\text{Standard Section Span})}{\text{Actual Section Span}}$$

The actual span for each cingulate segment was determined by the intersection interval multiplied by number of sections sampled. Sections were chosen for analysis using systematic random sampling. On a given coronal section, the cingulate gyrus was identified by its position on the medial aspect of the cerebral cortex, immediately above the corpus callosum and below the superior frontal gyrus. In Neurolucida or Stereo Investigator software, a contour was drawn around the entire pial surface of the cingulate. The lateral border of the contour was defined by the base of the cingulate sulcus extended perpendicularly to the corpus callosum. The boundary between the cingulate cortex and white matter was delineated using CTIP2 fluorescent staining in specimens younger than 5 months and NeuN fluorescent staining in specimens 5 months and older.

DCX, DAPI, and NeuN population sizes within a cingulate segment were estimated using the Optical Fractionator probe in Stereo Investigator. Unless stated otherwise, one brain was analyzed per age (N=1). Parameters for the Optical Fractionator study are given in Table S3. 3-

micron guard zones were used, along with counting frame dimensions of 50x50 microns for DAPI and DCX and 75x75 microns for NeuN. 3 to 6 sections were analyzed per brain. Range of sampling sites per brain: DAPI (cortex), 70-260; DAPI (white matter), 50-105; NeuN, 130-330; DCX: 325-930. Individual cells were counted using standard stereologic methods with a 63x oil immersion lens on a Zeiss Axioscope II epifluorescent microscope. Adequate sampling was monitored by calculating coefficients of error (Gundersen m=1), with all C.E.'s <0.1.

Interneuron subtype cell numbers in a cingulate segment were estimated using an exhaustive counting approach, as their scarcity at certain ages and in some regions (e.g. white matter) made an Optical Fractionator study impractical. Cells were counted exhaustively on tiled images of fluorescently labeled sections, acquired at 10x magnification. Neurolucida was used to keep track of both the total number of cells counted and their spatial distribution. Total interneuron cell number per cingulate segment was then calculated by summing the exhaustive cell counts across all sections and then correcting for the section sampling fraction and normalizing to a standardized segment of 150 sections, as described above. 3 to 6 sections were used per brain. Section sampling frequencies for subtype counts are given in Table 4.

For counts on the subpallial transcription factors near the ventricular wall and within the Arc, 3 equivalent fields of view were imaged for each region, using a 40x lens on a Leica white light confocal microscope, and quantified. 3-4 individual neonatal cases were used for a total of 9-12 samples per count.

Calculation of cingulate volumes. The Cavalieri Estimator in Stereo Investigator was used to calculate the volume of cingulate segments at each age. Cingulate segments were collected as described above for cell quantifications and as depicted in fig. S9A. Ten sections, spaced 15 sections apart were chosen for analysis. Mounted section thickness was 30 microns. Grid size was 500 microns. Coefficients of error (Gundersen m=1) were <0.04 for all specimens analyzed.

Organotypic slice cultures and live imaging. When autopsy was performed with a post-mortem interval less than 18hrs, tissue was collected for live-imaging experiments. The fresh, unfixed, tissue was cut either in a coronal or sagittal block that was less than 1cm thick. It was kept in cold artificial cerebrospinal fluid (ACSF) that was oxygenated until embedded in 3.5% low-melting-point agarose (Fisher) and sectioned using a Leica VT1200S vibrating blade microtome to 250-300μm slices in ACSF containing 125 mM NaCl, 2.5 mM KCl, 1 mM $MgCl_2$, 1 mM $CaCl_2$ and 1.25 mM NaH_2PO_4. The sections were then transferred to Millicell-CM slice culture inserts (Millipore) that were immersed in cortical slice culture medium [66% BME, 25% Hanks, 5% FBS, 1% N-2, 1% penicillin, streptomycin, and glutamine (all Invitrogen) and 0.66% D-(+)-glucose (Sigma-Aldrich)]. An adenovirus (AV-CMV-GFP, 1×10^{10}; Vector Biolabs) at a dilution of 1:50–1:500 was applied to the slices, which were then cultured at 37°C, 5% CO_2, 8% O_2. For time-lapse imaging, cultures were then transferred to an inverted Leica TCS SP5 confocal microscope with an on-stage incubator streaming 5% CO_2, 5% O_2, and balanced N_2 into the chamber. Slices were imaged using a 10× air objective at 25 min intervals for up to 3 d with repositioning of the *z*-stacks every 6-8h. For *post hoc* analysis, slices were fixed in 4% PFA and processed as floating sections for immunohistochemistry as described above.

MRI. The images were acquired on a full body GE MR950 7T scanner using a 32-ch Nova Medical head coil with 3D fast spin echo sequence, isotropic 600-micron resolution, time to echo (TE) of ~120ms and TR of 2.5s, and 8 averages. The scan time was approximately 30 minutes. The distribution of migratory cells were manually labeled using MNI-Display software (http://www.bic.mni.mcgill.ca/ServicesSoftwareVisualization/Display). The cells on the MRI images appear distinctively darker than white matter intensity, and look similar to the intensity of gray matter. Previous studies had recognized a periventricular signal that was distinct from that of the overlying developing white matter (*44, 45*). The rater works mainly on the sagittal plane, and trimmed or added segmentation using the axial and coronal views if necessary. One rater (M.P.) segmented all cases and reviewed the resulting labels with H.K. The rater repeated segmentation with a one-month time distance. The intra-rater reproducibility was excellent as the Dice overlap index (ref) was 89±1%.

Quantitative analyses on MRI. We measured two different characteristics of the migratory cell distribution: extent and location of the cell distribution. To measure the extent, we first skeletonized the whole label using a mathematical solution developed previously (*46*). This algorithm further created a distance map from one end to the other along the skeleton. The maximum distance between the two ends of the skeleton was normalized by dividing it with the whole extent of the ventricle, and was used for the measurement of extent. Regarding the location of the cell distribution, we were interested in how the distribution was located with respect to the lateral ventricle in the anterior-posterior direction. To assess this for each individual, we computed the centroids of the label volume and of the lateral ventricle. We computed the distance between these two centroids in y-axis (i.e., anterior-posterior direction),

and normalized it using the distance between the anterior and posterior tips of the ventricle. We used this normalized value to assess the location of cell distribution.

Electron microscopy. For transmission electron microscopy (TEM), samples were sectioned with a vibrating blade microtome (200 microns), postfixed with 2% osmium tetroxide solution. Sections were dehydrated in crescent ethanol concentrations and stained with 2% uranyl acetate, and finally embedded in araldite resin (Durcupan ACM Fluka, Sigma), and allowed to solidify at 37°C for 72 hours. Ultrathin sections were obtained (70 nm) and were contrasted with lead citrate solution on the grids. Pre-embedding immunohistochemistry was performed on 50 microns floating sections with DCX and GFAP antibodies and developed with diaminobenzidine (DAB). Postfixation was performed with 7% glucose-1% Osmium tetroxide and, afterwards, followed a conventional embedding protocol.

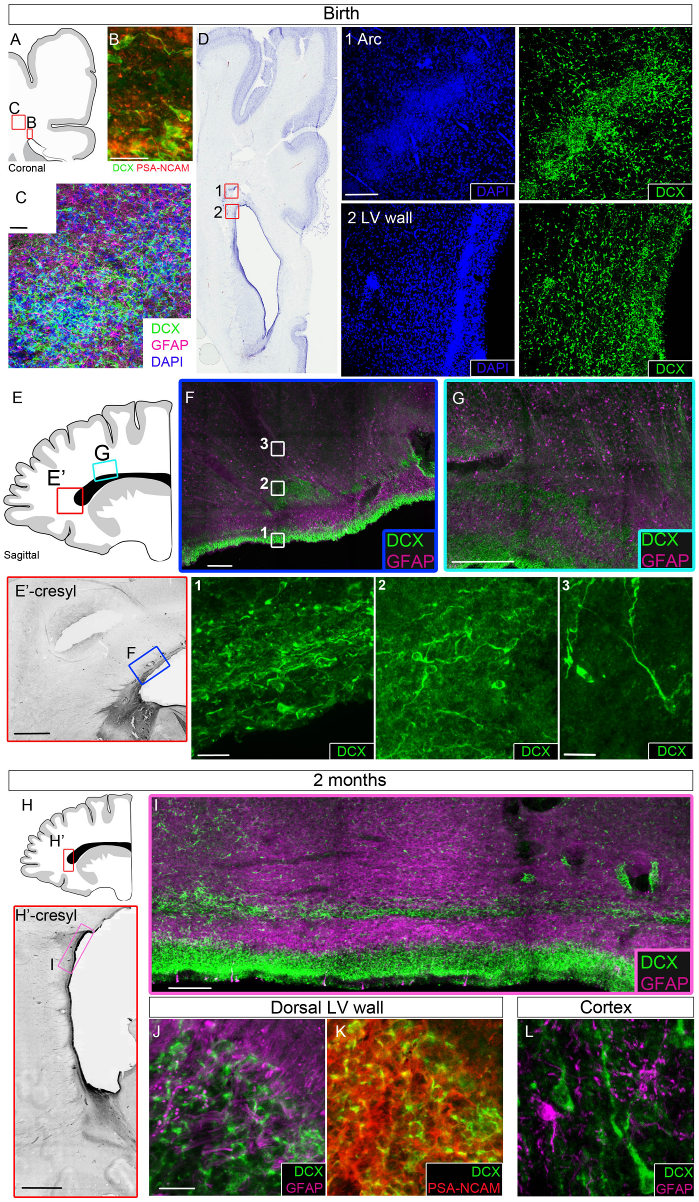

Figure S1. Evidence of a collection of young migratory neurons, the Arc, in the infant brain. (**A-B**) Dense collections of DCX+PSA-NCAM+ cells are observed in coronal sections at the dorsolateral edge of the lateral ventricle (LV) at birth. (**C**) DCX+ are cells surrounded by GFAP+ cells and fibers. (**D**) Nissl-stained collections of cells in the Arc (1) and in the ventricular and subventricular zones of the LV wall (2); to the right are higher magnification images of DAPI and DCX-stained cells in fields in (D). (**E-G**) Sagittal sections showing the

tiered organization (see text) of DCX+ cells around the ventricular wall at anterior (F) and more posterior (G) levels. Higher magnification image of this region showing DCX+ cells (1) densely collected at the SVZ, (2) as a separate strip of clustered cells, and (3) individually dispersed and oriented towards the pia. (**H** and **I**) At two months, the cellular densities have thinned out (H) but are still composed of DCX+ cells (I). (**J** and **K**) The SVZ region remains enriched with DCX+PSA+ cells and the tiered levels are still present. (**L**) Within the overlying gray-white matter border, we can identify many DCX+ cells radially oriented towards the pia. Of note, the brains shown here, at birth and 2 months, were previously scanned with 7T MRI. Scale bars, 25 microns (B); 100 microns [(C) and (D)]; 200 microns (E); 300 microns (F); 500 microns (G), 25 microns (inset); 150 microns [(H) to (I)]; 25 microns [(J) to (L)].

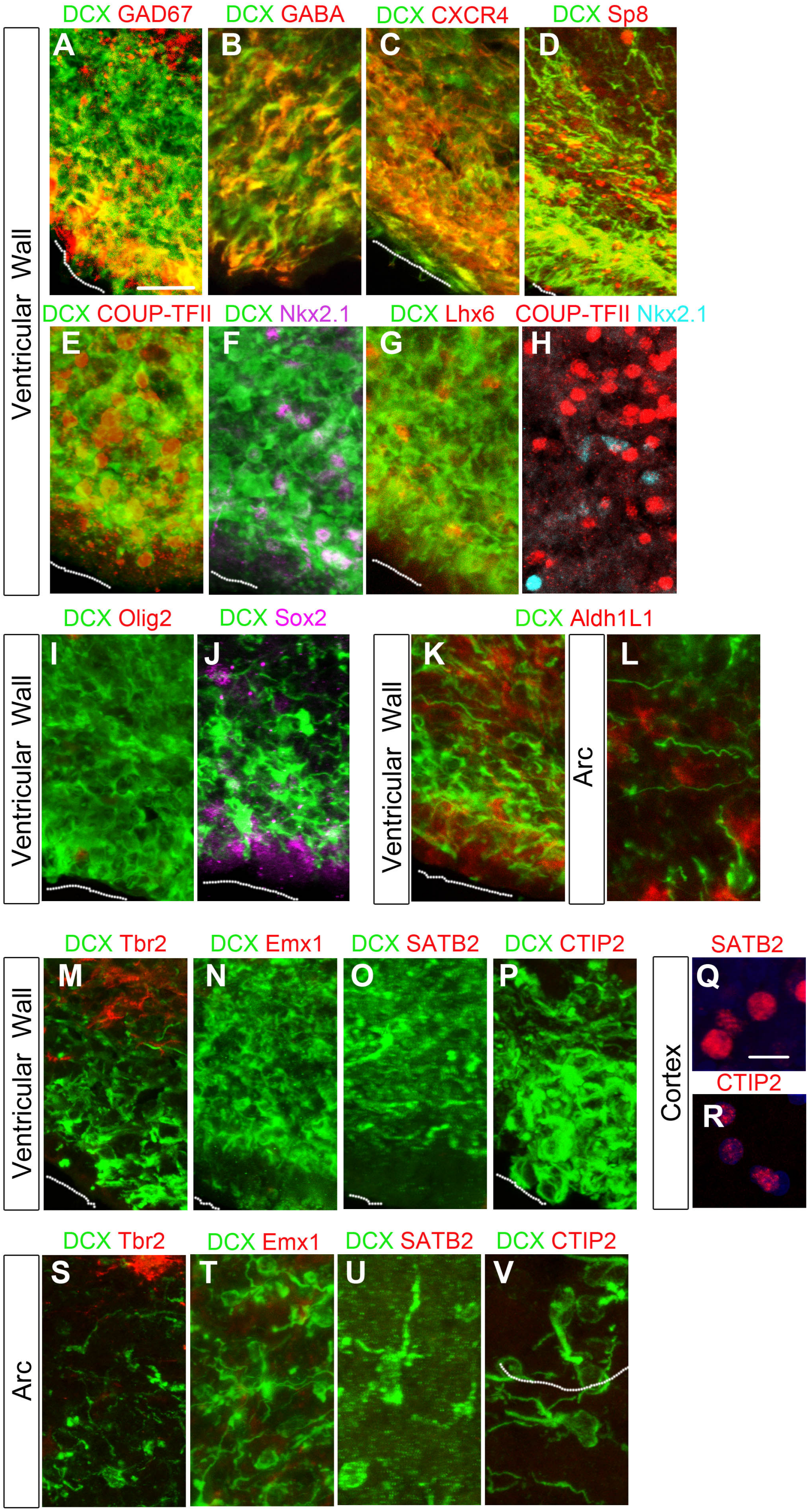

DCX GAD67
DCX GABA
DCX CXCR4
DCX Sp8
A
B
C
D
Ventricular Wall
DCX COUP-TFII
DCX Nkx2.1
DCX Lhx6
COUP-TFII Nkx2.1
E
F
G
H
DCX Olig2
DCX Sox2
DCX Aldh1L1
I
J
K
L
Ventricular Wall
Ventricular Wall
Arc
DCX Tbr2
DCX Emx1
DCX SATB2
DCX CTIP2
SATB2
M
N
O
P
Q
CTIP2
R
Ventricular Wall
Cortex
DCX Tbr2
DCX Emx1
DCX SATB2
DCX CTIP2
S
T
U
V
Arc

Figure S2. Expression profile of migrating DCX+ cells in the infant brain. (**A**-**C**) DCX+ cells at the dorsal ventricular wall express GAD67, GABA, and CXCR4, a receptor seen in migrating interneurons. (**D**-**G**) A subpopulation of the DCX+ cells were SP8+, COUP-TFII+, Nkx2.1+, or Lhx6+. (**H**) COUP-TFII+ and Nkx2.1+ cells did not overlap. (**I**-**L**) DCX+ cells do not express Olig2, Sox2, or AldhL1. DCX+ cells at the dorsal ventricular wall or the Arc do not express Tbr2 (**M** and **S**), associated with intermediate precursors, or Emx1, SATB2, and CTIP2 (**N-P**; **T-V**), transcription factors in excitatory neurons derived from dorsal progenitor regions. (**Q**-**R**) SATB2 and CTIP2 expression is seen in the cortex, as would be expected. Dashed lines show the border of the lateral ventricle wall. Scale bars, 20 microns [(A) to (P) and (S) to (V)]; 10 microns [(Q) to (R)].

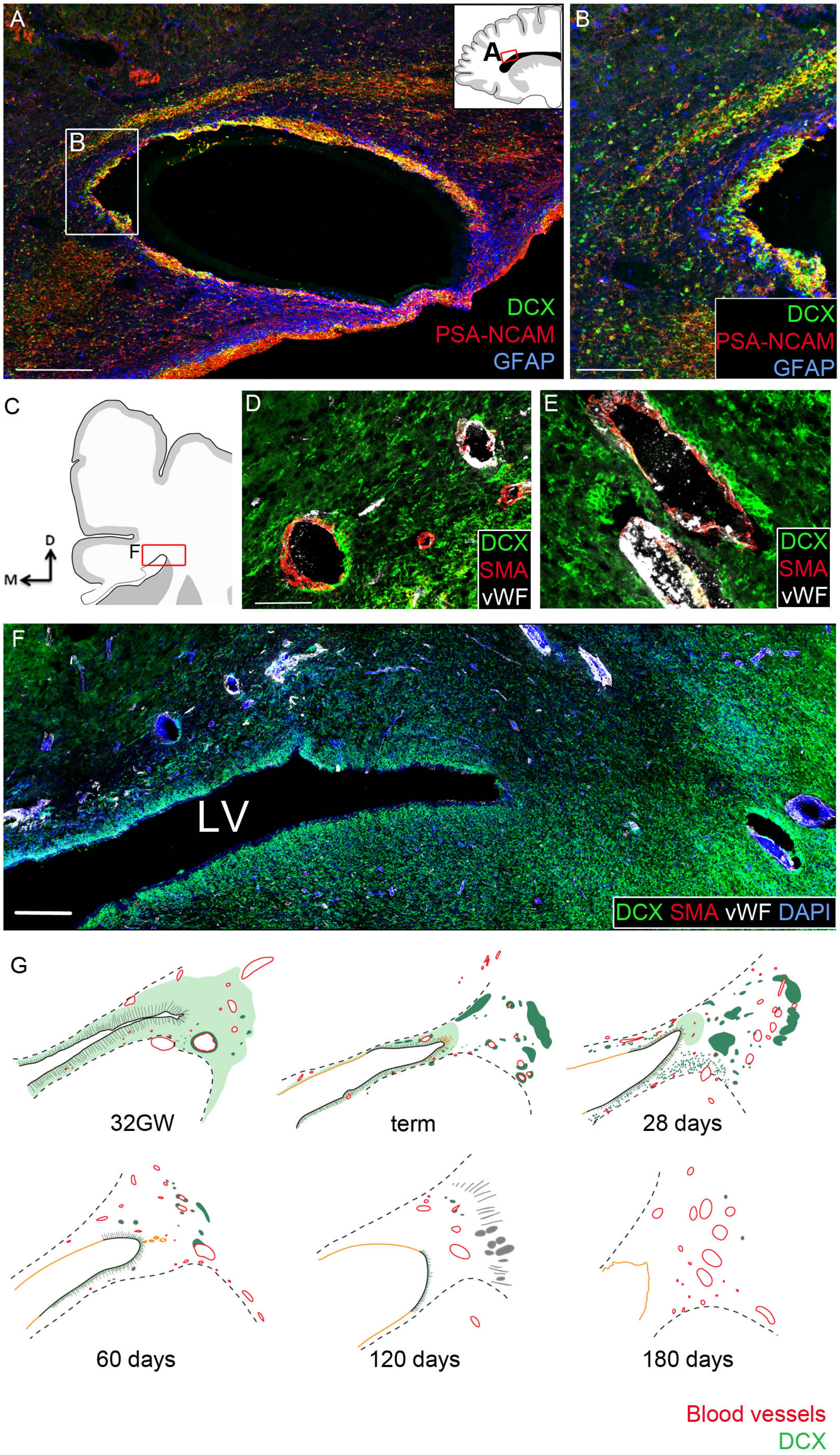

A
B
DCX
PSA-NCAM
GFAP
C
D
M
F
E
SMA
vWF
LV
DAPI
G
32GW
term
28 days
60 days
120 days
180 days
Blood vessels
DCX

Figure S3. DCX+ cells cluster around blood vessels in Tier 3. (**A-B**) DCX+PSA+ cells were intimately associated with blood vessels. (**C-F**) Arteries around the dorsolateral edges of the ventricle, labeled by vonWillebrands factor (vWF) and smooth muscle actin (SMA), were densely surrounded by DCX+ cells. (**G**) The close relationship between DCX+ cells and blood vessels decreases by 60 days after birth, and DCX+ cells have decreased in this region by 120 days after birth. Scale bars, 250 microns [(A) and (F)]; 100 microns [(B) to (E)].

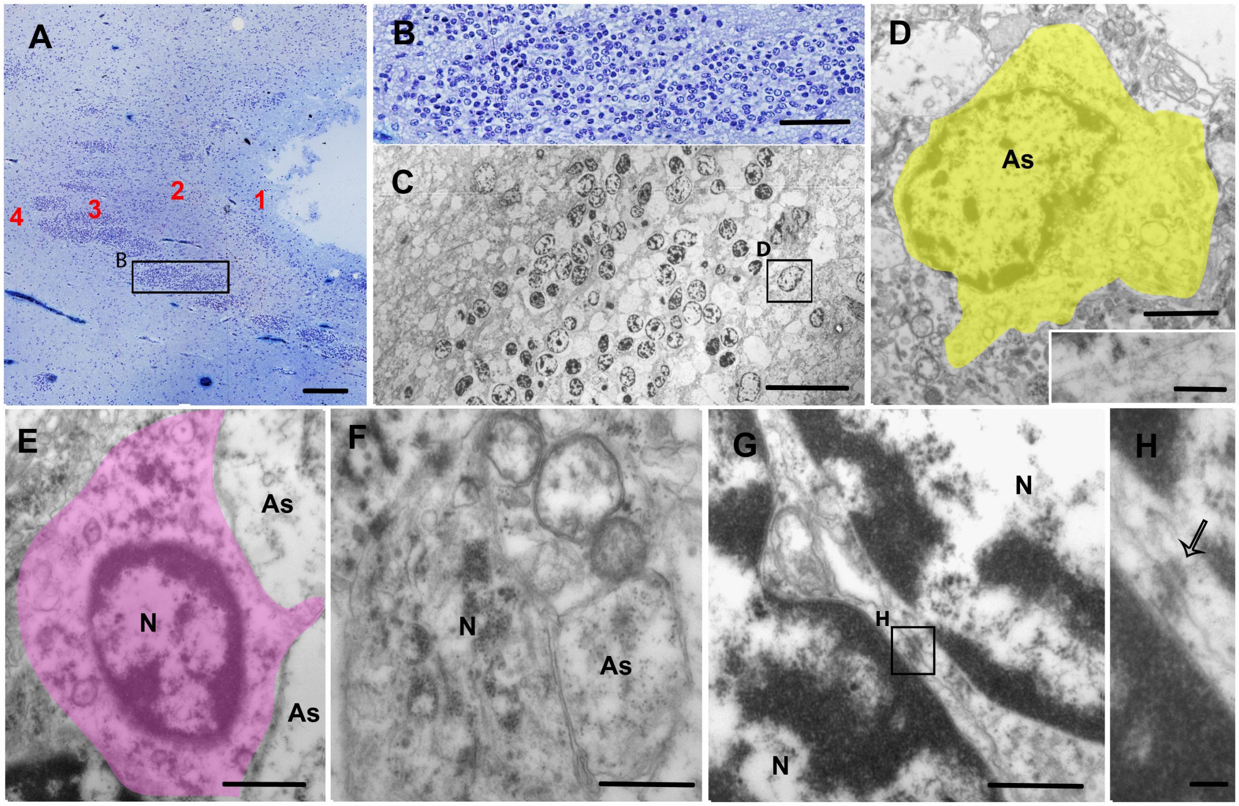

Figure S4. Electron Microscopy of the Arc at birth. (**A**) Panoramic sagittal view of the Arc in semithin sections stained with toluidine blue. The Arc is composed of multiple chains that converge rostrally. Tiers 1-4 (refer to main text) are numbered. (**B**) Higher magnification of the boxed inset in (A) shows the organization of individual chains in more detail. (**C**) Ultrastructual analysis of an individual chain with flanking astrocytes; rostrally the astrocytic sheath is less prominent and the clusters are not densely wrapped. (**D**) The parenchymal astrocyte (As) flanking the chain is characterized by large, round nuclei with peripheral heterochromatin. The cytosol has scarce organelles and occasional bundles of intermediate filaments (inset in D). (**E**) A representative young neuron (N) within the chain has a small nucleus and very condensed chromatin. There is a thin rim of cytosol without intermediate filaments. Microtubules and

ribosomes are observed as dense granular bodies; this is present in the astrocytic soma or expansions. Two light glial expansions (As) are seen to the right of the neuron. (**F**) Expansions within the chain are light (associated with astrocytes) or contain dense granular structures (associated with young neuron). (**G**) Two neuronal cells can occasionally make contact via dense cell-to-cell adhesions. This is shown in more detail in (**H**). Scale bars, 500 microns (A); 100 microns (B); 25 microns (C); 2 microns (D), detail 50nm; 2 microns (E); 5 microns (F); 1 micron (G); 5 nm (H).

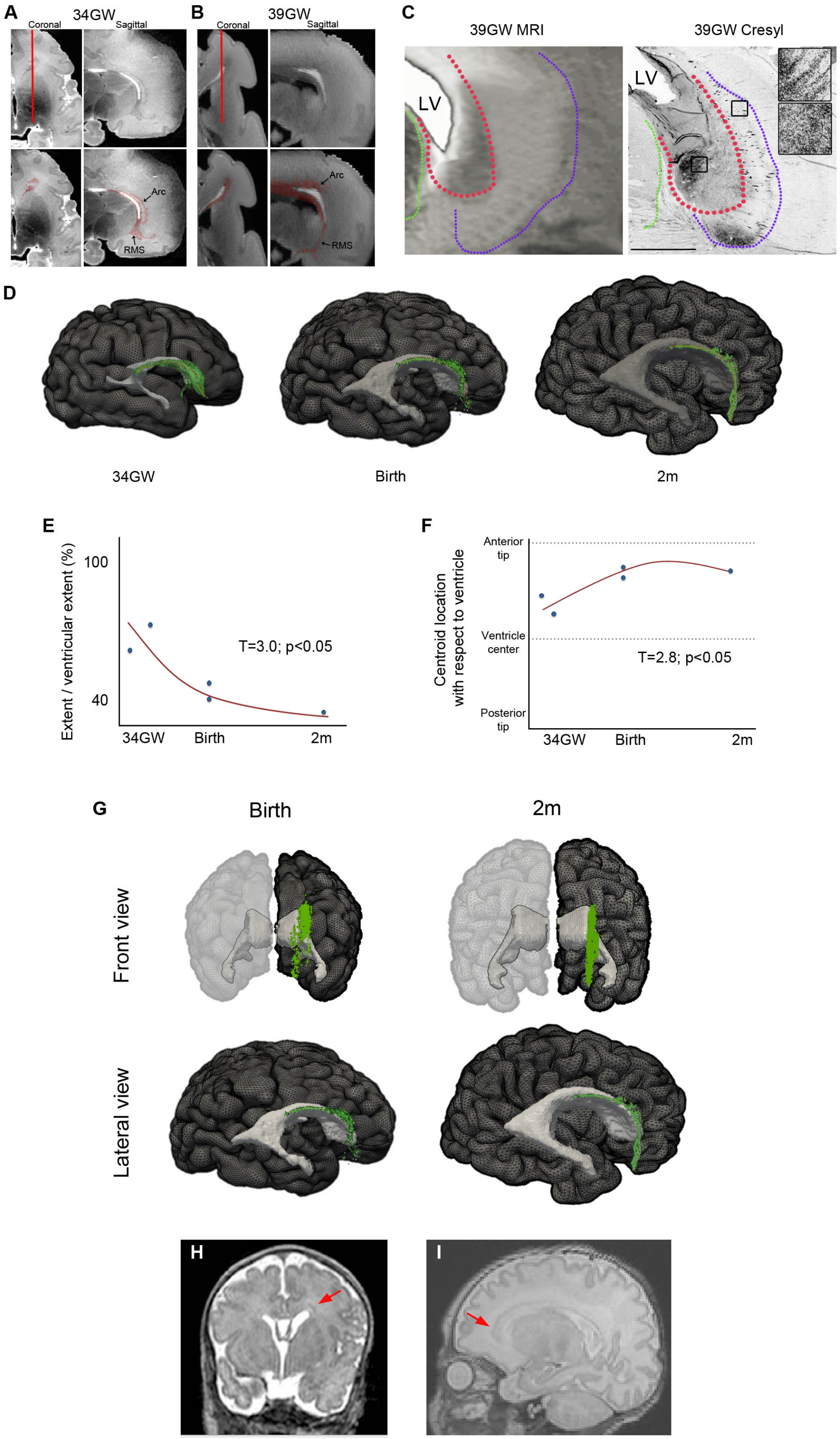

A
34GW
Coronal
Sagittal
Arc
RMS
B
39GW
Coronal
Sagittal
Arc
RMS
C
39GW MRI
39GW Cresyl
LV
LV
D
34GW
Birth
2m
E
Extent / ventricular extent (%)
100
40
T=3.0; p<0.05
34GW
Birth
2m
F
Centroid location with respect to ventricle
Anterior tip
Ventricle center
Posterior tip
T=2.8; p<0.05
34GW
Birth
2m
G
Birth
2m
Front view
Lateral view
H
I

Figure S5. High-resolution imaging of infant brains reveals a collection of cells around the anterior body of the lateral ventricle. (**A** and **B**) Representative images of T2 sequences (top panels) scanned at 34GW and birth (39GW). Bottom panels show the associated segmentation signal in orange. The segmentation signals correspond anatomically to the Rostral Migratory Stream (RMS) and, more dorsally, to the dorsolateral cells clusters that make up the Arc. Red lines in coronal images indicate the medial-lateral level shown in sagittal images. (**C**) MRI and corresponding cresyl violet staining of sagittal sections from a postmortem neonatal (39GW) frontal lobe. Both images are taken at the anterior head of the lateral ventricle (LV). The T2 hyperintense signals in the MRI correspond to regions of high cell density seen in the cresyl staining. Dense cell collections at the ventricular wall are outlined in red, and clusters further removed are outlined in purple. Insets of the cresyl stained section show the dense cell clusters at these two locations. (**D**) Lateral view of 3D rendering of brain MRI at 34GW, birth, and 2 months shows the segmentation of T2 intensity associated with the Arc in green. The skeletal analysis (see quantitative MRI methods) for measurements is shown as a dashed yellow line in each brain. (**E**) Quantification of cell distribution length (extent) relative to ventricular length (exent). (**F**) Location of center of segmentation volume corresponding to the Arc (centroid) with respect to lateral ventricle. (**G**) Postmortem brains were scanned on 7T MRIs at birth and 2 months. T2 signals associated with the Arc are displayed in green and are shown in both frontal and lateral perspectives. (**H** and **I**) 3T MRI images of 2 pediatric patients taken at 34GW and 33GW show that the T2 signal corresponding to the Arc (red arrows) could be visualized in live MRI. Scale bar, 150 microns.

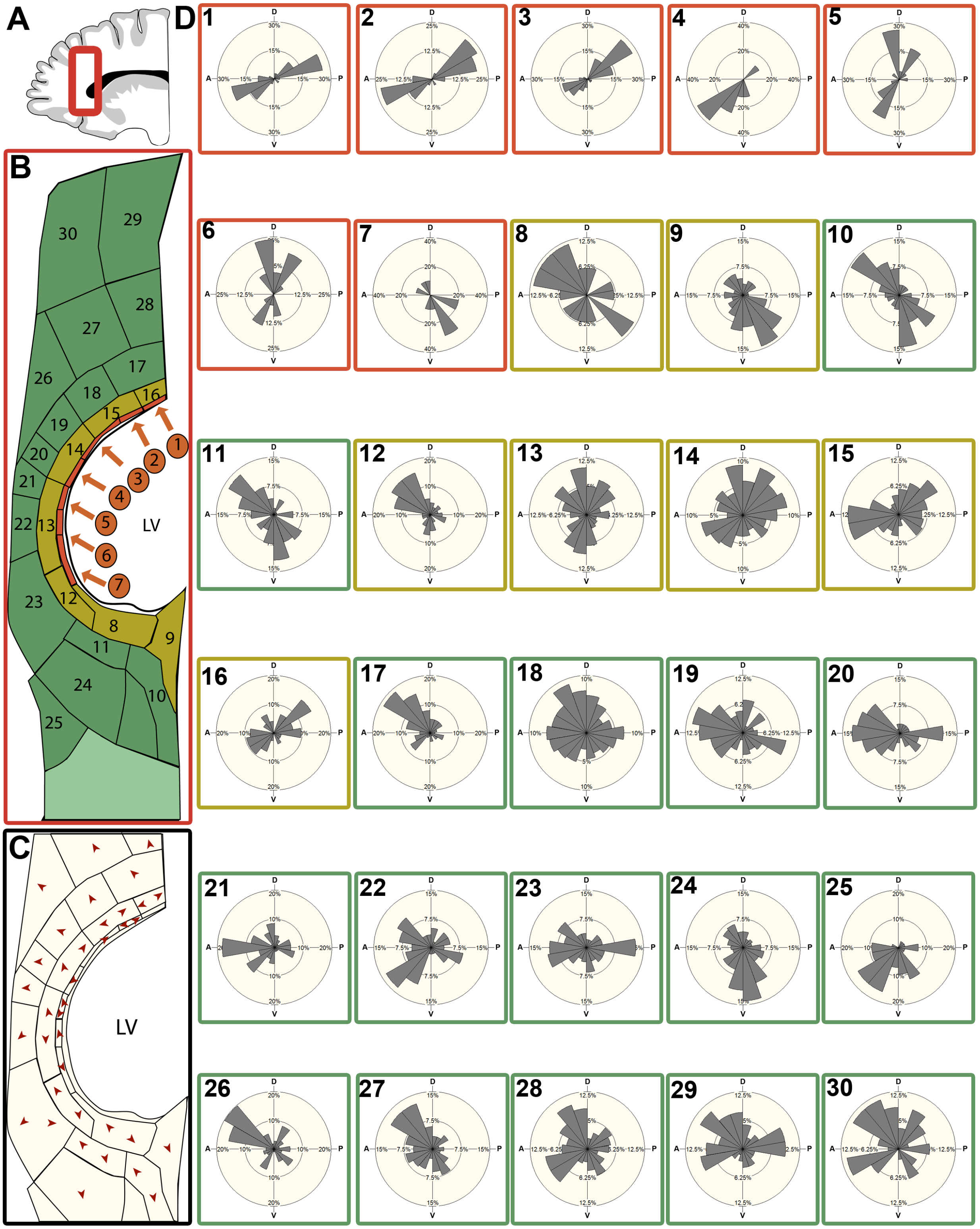
A
B
C
D
LV

Figure S6. Directionality of DCX+ cells at birth (sagittal view). (**A**) Red box indicates region depicted in (B). (**B**) Schematic diagram indicating the location of each numbered subregion analyzed. The directionality of at least 50 cells was determined within each subregion. (**C)** Red arrows indicate the modal (most frequent) direction of DCX+ cells' leading process. (**D**) Directional histograms for each subregion indicate the percentage of cells oriented in a given direction. Histogram axis labels: A, anterior; P, posterior; D, dorsal; V, ventral.

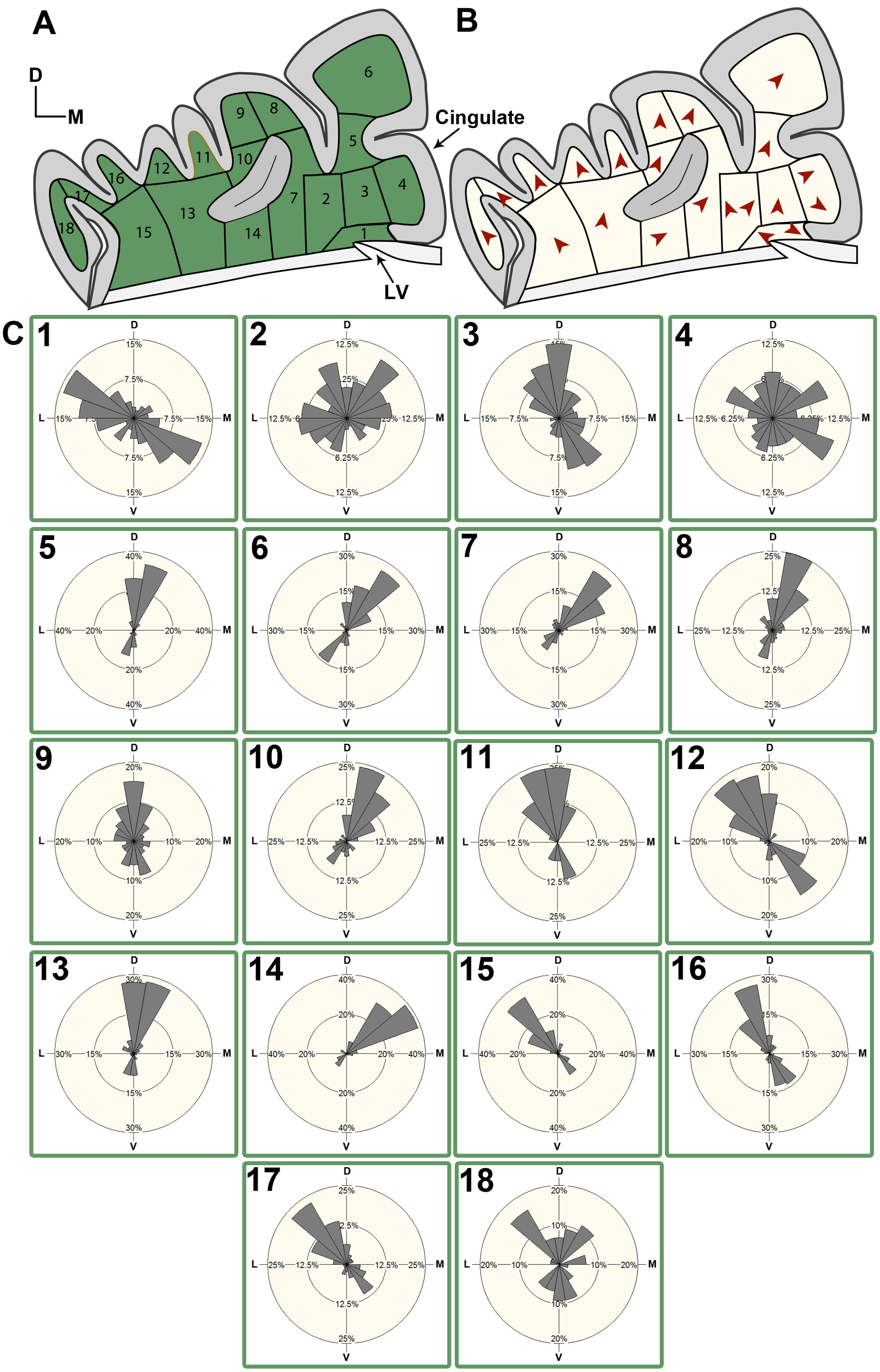
A
B
C
D
M
Cingulate
LV
1
2
3
4
5
6
7
8
9
10
11
12
13
14
15
16
17
18

Figure S7. Directionality of DCX+ cells at 10 days after birth (coronal view). (A) Schematic diagrams indicating the location of each numbered subregion analyzed. The directionality of at least 50 cells was determined within each subregion. (**B**) Red arrows indicate the directional mode for subregions. (**C**) Directional histograms for each subregion indicate the percentage of cells oriented in a given direction. Histogram axis labels: L, lateral; M, medial; D, dorsal; V, ventral.

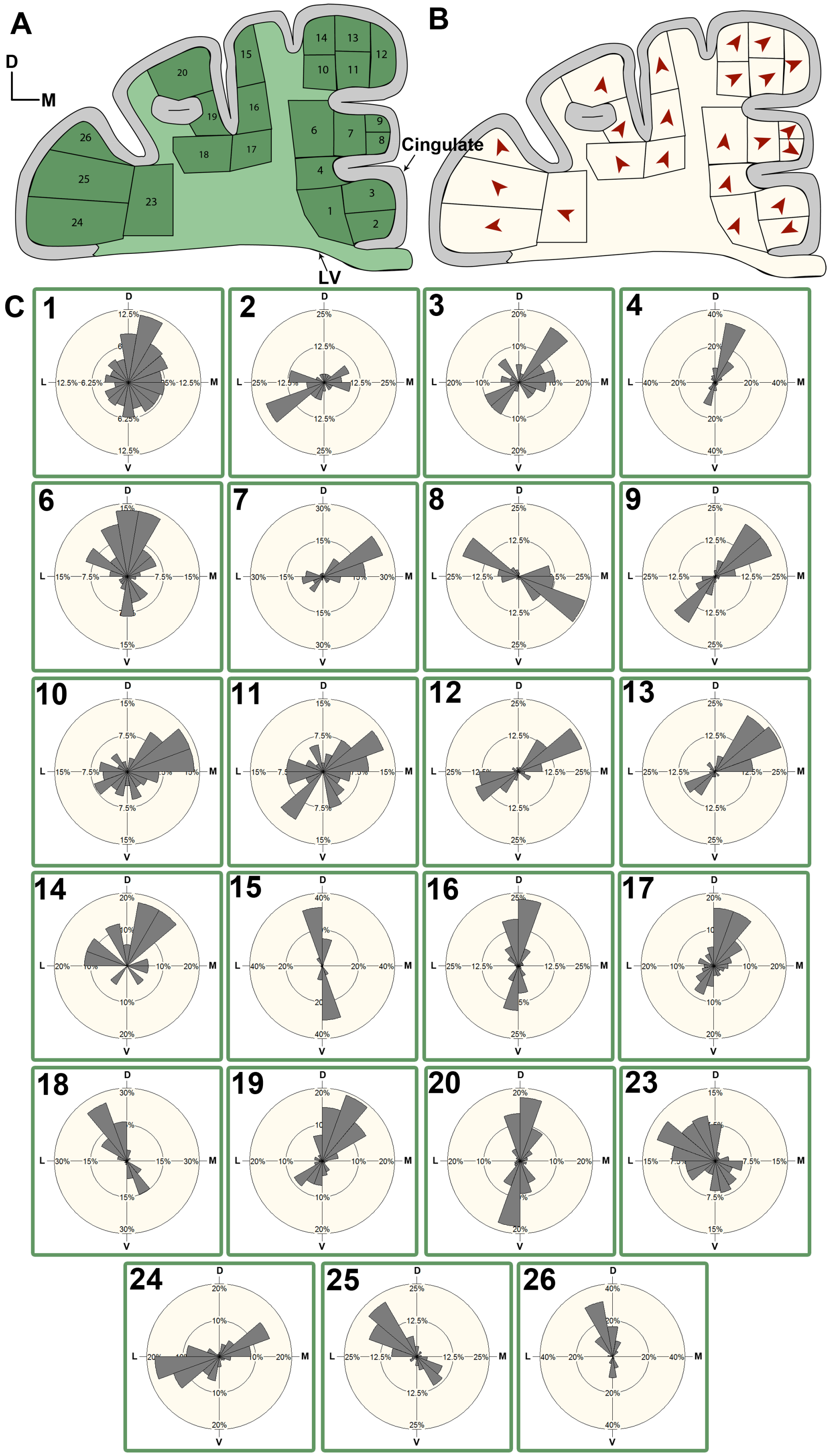

A
B
C
D
M
Cingulate
LV

Figure S8. Directionality of DCX+ cells at 1.5 months after birth (coronal view). (A) Schematic diagrams indicating the location of subregions analyzed. The directionality of at least 50 cells was determined within each subregion. (**B**) Red arrows indicate the directional mode (most frequent direction) for each subregion. (**C**) Directional histograms for each subregion indicate the percentage of cells oriented in a given direction. Histogram axis labels: L, lateral; M, medial; D, dorsal; V, ventral.

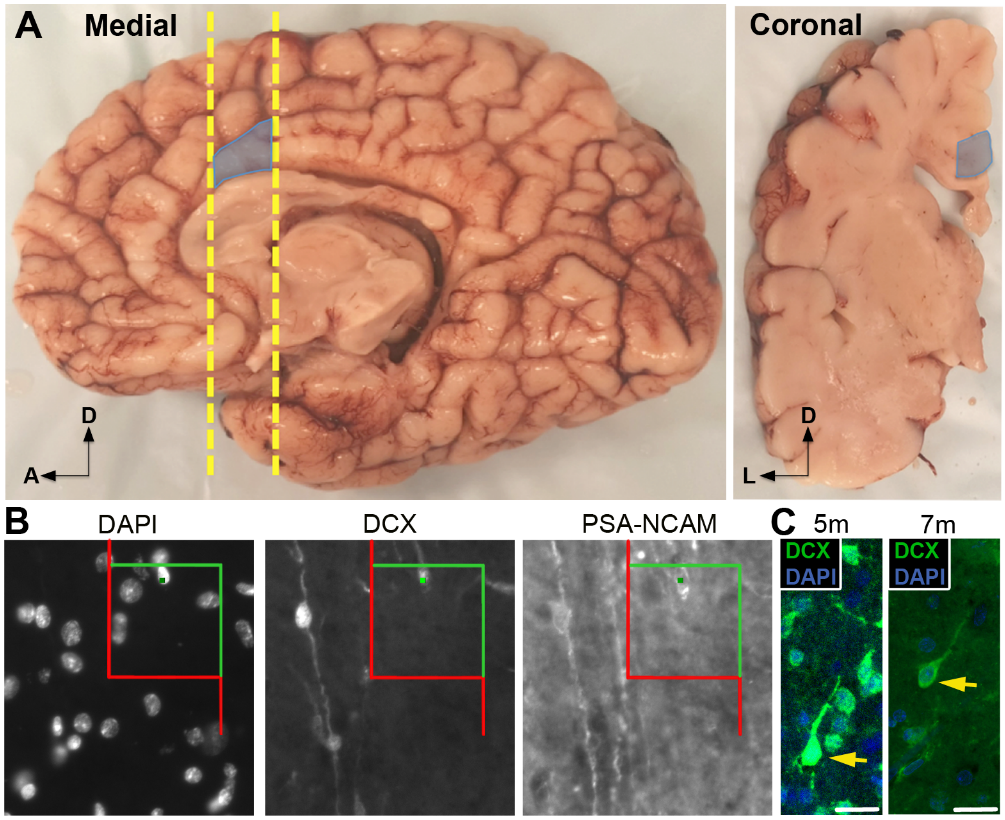

Figure S9. Stereological quantification of DCX+ cells in the cingulate gyrus. (**A**) Example of a neonatal brain used for all stereology studies. Dashed yellow lines indicate where a coronal cut is made. Shaded blue region between the dashed lines corresponds to a "cingulate segment." Coronal sections within the "cingulate segment" are used for quantification. (**B**) Example of a DCX+/PSA-NCAM+ cell at 10 days after birth that was counted using the Optical Fractionator probe. (**C**) Representative immunolabeling of young migratory neurons in the cingulate cortex at 5 months and 7 months. Cells that are considered migratory are indicated by yellow arrows. Scale bars, 20 microns. Directional axes: A, anterior; D, dorsal; L, lateral.

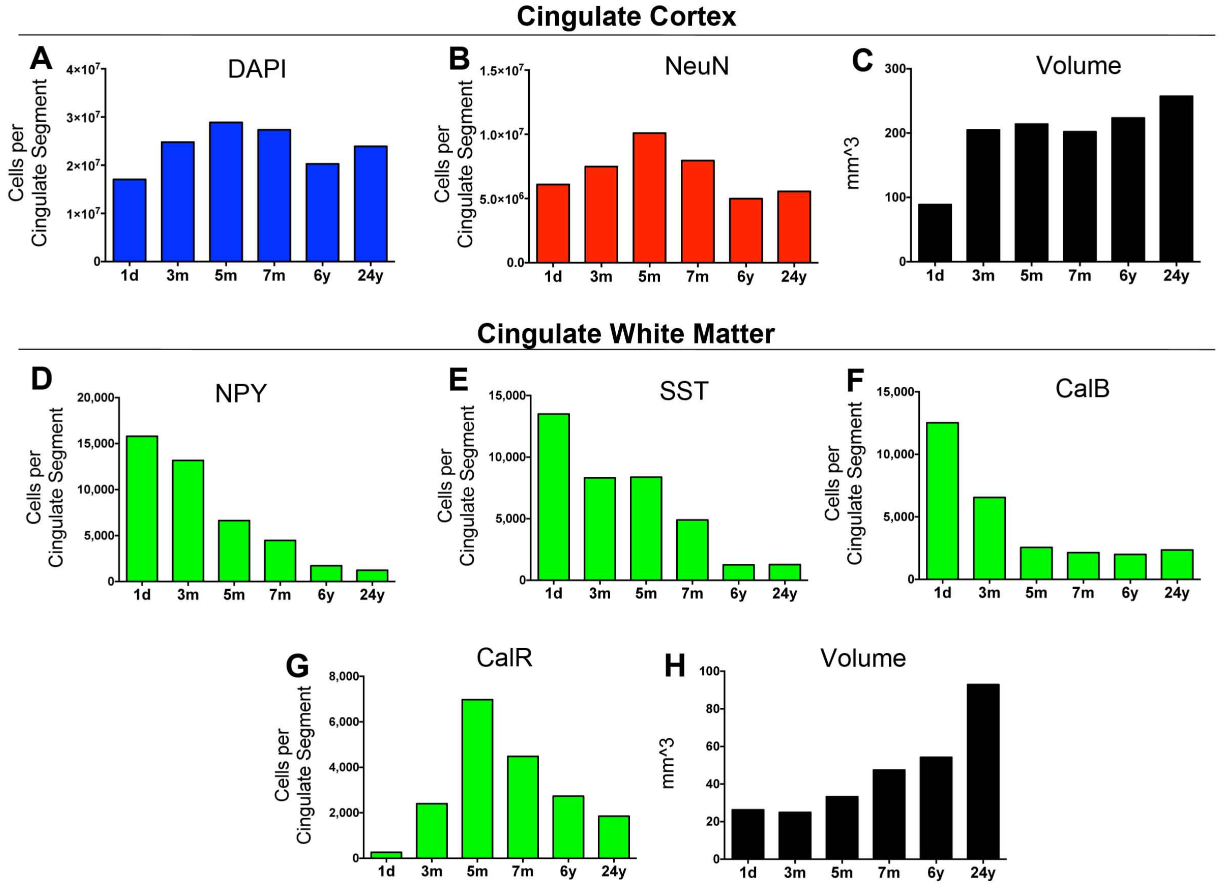

Figure S10. Quantification of cellular populations in the cingulate gyrus. The total number of cells (**A**) and neurons (**B**) within the cingulate cortex increases during early postnatal life. In the cingulate white matter, dramatic changes occur in the sizes of interneuron subtype populations: the number of NPY (**D**), SST (**E**), CalB (**F**) cells decrease as these cells are possibly pruned or move to the cortex; CalR (**G**) increases then decreases. The volume of the cortex (**C**) and white matter (**H**), as measured by the Cavalieri Estimator, also increases after birth: cortical volume increases but remains relatively stable after 3 months, while white matter volume increases dramatically by 24 years, possibly due to oligodendrocyte proliferation and myelination.

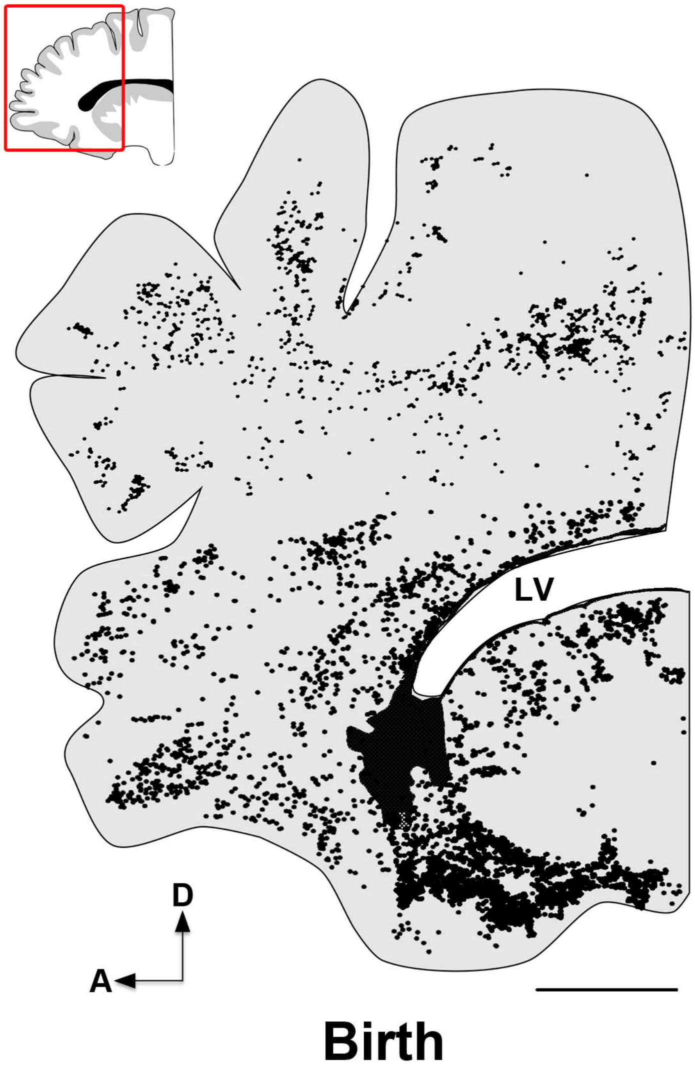

Figure S11. Anterior distribution of DCX+ cells in the human frontal lobe. In a sagittal section of the frontal lobe at birth, DCX+ cells with migratory morphology are widely distributed throughout the developing white matter, cingulate gyrus and prefrontal cortex. Black dots correspond to individual DCX+ cells with migratory morphology. Scale bar, 10.5 millimeters. Directional axes: A, anterior; D, dorsal.

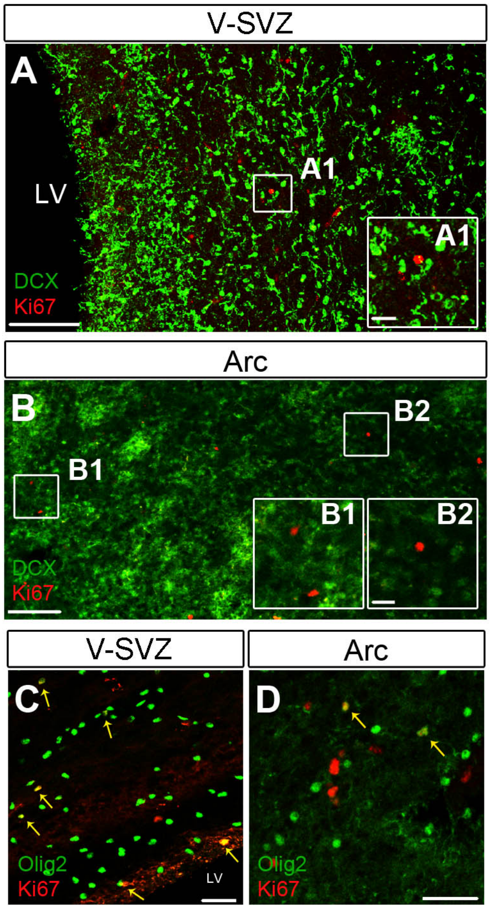

Figure S12. Proliferation at the dorsal ventricular wall at birth. Few cells express Ki67, a marker of proliferation, at the dorsal ventricular wall or in the clusters within the Arc. There was no co-expression of DCX with Ki67 (**A-B**), but many of the Ki67+ cells were found to be Olig2+ (**C-D**). Scale bars, 100 microns [(A) and (B)] with 20 microns in insets; 100 microns (B); 75 microns (C); 100 microns (D).

Table S1. Clinical and experimental demographics of collected human specimens in this study

Case Number	Age	Gender	Clinical Diagnosis	Neuropathology Diagnosis	Experimental Use
1	34GW	M	Omphalocele	control	histology and IHC
2	34GW	M	cloacal anomaly	control	histology and IHC and MRI
3	34 GW	M	coarctation of aorta; PHACE	control	histology and IHC
4	37 GW	M	placental eruption	control	histology and IHC
5	38 GW	M	cardiac arrest	control	histology and IHC
6	38 GW	F	congenital cardiovascular disorde	control	histology and IHC
7	39 GW	F	intrauterine demise	control	histology and IHC
8	39 GW	F	TE fistula; pulmonary hypoplasia	control	histology and IHC and electron microscopy
9	0 day	M	diaphragmatic hernia	control	histology and IHC and MRI
10	0 day	M	pulmonary fibrosis	control	histology and IHC, time-lapse imaging, and MRI
11	0 day	M	multicystic kidney disease	control	histology and IHC
12	0 day	F	renal agenesis	control	histology and IHC
13	0 day	M	cardiac anomaly	control	histology and IHC
14	0 day	F	situs inversus	control	time-lapse imaging and histology and IHC
15	0 day	M	hypoplastic heart complex	control	histology and BV analysis
16	1 day	M	chondrodysplasia	control	histology and IHC
17	1 day	M	sepsis	control	histology and IHC
18	1 day	M	diaphragmatic hernia	control	histology and IHC
19	2 days	F	bronchopneumonia	control	histology and IHC
20	2 days	F	sepsis	control	histology and IHC, time-lapse imaging, and MRI
21	1 week	M	acute renal failure	control	histology and IHC and MRI
22	10 days	F	atypical motor neuron disease	control	histology and IHC
23	2 weeks	F	cardiopulmonary failure	control	histology and IHC
24	3 weeks	M	pulmonary fibrosis	control	histology and IHC
25	28 days	M	SIDS	control	histology and electron microscopy
26	30 days	M	sepsis	control	histology and BV analysis
27	40 days	F	diaphragmatic hernia	control	histology and IHC
28	1.5 months	F	congenital connective tissue diso	control	histology and IHC
29	2 months	M	SIDS	control	histology and BV analysis
30	2 months	F	bronchopulmonary dysplasia	control	histology and IHC, time-lapse imaging, and MRI
31	3 months	M	diaphragmatic hernia	control	histology and IHC
32	3 months	M	coarctation of aorta	control	histology and IHC
33	4 months	F	sepsis	control	histology and Bv analysis

Table S1. Clinical and experimental demographics of collected human specimens in this study

34	5 months	M	diaphragmatic hernia	control	histology and IHC
35	5 months	F	coarctation of aorta	control	histology and IHC and MRI
36	6 months	F	SIDS	control	histology and BV analysis
37	7 months	M	Wisott-Aldrich syndrome	control	histology and IHC
38	8 months	M	Sepsis	control	histology and BV analysis
39	2 years	M	leukemia	control	histology and IHC
40	6 years	F	lung hypoplasia	control	histology and IHC
41	14 years	F	anorexia	control	histology and IHC
42	15 years	M	leukemia	control	histology and IHC
43	19 years	M	biliary atresia	control	histology and IHC
44	24 years	F	hepatic failure, Crohn's	control	histology and IHC
45	41 years	F	pancreatitis	control	histology and IHC
46	49 years	F	non-CNS tumor	control	histology and IHC
47	52 years	M	cavernous malformation	control	histology and IHC
48	57 years	F	papillary thyroid carcinoma	control	histology and IHC
49	67 years	F	COPD	control	histology and IHC
50	74 years	M	non-CNS tumor	control	histology and IHC

Table S2. Primary Antibodies Used

Primary Ab	Species	Dilution	Manufacturer	Cat. No.	Antigen Retrieval
Calbindin	Rabbit	1:750	Swant	CB-38a	10 min
Calretinin	Mouse	1:750	Swant	6B3	10 min
CoupTF2	Mouse	1:100	R&D Systems	PP-H7147-00	11 min
Ctip2	Rat	1:500	Abcam	ab18465	None
CXCR4	Rabbit	1:100	Abcam	ab124824	9 min
Doublecortin	Rabbit	1:200	Cell Signaling	4604	None
Doublecortin	Guinea Pig	1:200	Millipore	ab2253	None
Emx1	Mouse	1:200	Santa Cruz	sc-398115	10 min
GABA	Rabbit	1:1000	Sigma Aldrich	A2052-.2ML	10 min
GAD67	Goat	1:500	Abcam	ab80589	30 min
GFAP	Chicken	1:750	Abcam	ab4674	None
LHX6	Mouse	1:200	Santa Cruz	sc-271433	10 min
NeuN	Chicken	1:500	Millipore	ABN91	None
Neuropeptide Y	Rabbit	1:750	Abcam	ab30914	10 min
Nkx2.1	Rabbit	1:100	Santa Cruz	SC-13040	11 min
Olig2	Rabbit	1:200	Millipore	AB9610	None
Parvalbumin	Rabbit	1:500	Swant	PV-25	8 min
PSA-NCAM	Mouse	1:500	Millipore	MAB5324	None
SATB2	Mouse	1:500	AbCam	ab51502	5 min
SMA	Mouse	1:500	Sigma	a2547	None
Sox2	Goat	1:200	Santa Cruz	SC-17320	11 min
Sp8	Goat	1:100	Santa Cruz	SC-104661	10 min
Somatostatin	Goat	1:200	Santa Cruz	sc-7819	10 min
Tbr2	Rabbit	1:200	Abcam	ab75720	8 min
vWF	Rabbit	1:400	Dako	A0082	5 min

Table S3. Optical Fractionator Parameters

**Section Sampling Fraction**	**Birth**	**10 days**	**42 days**	**3 months**	**5 months**	**7 months**	**6 years**	**24 years**
**DAPI**	1/40	Not analyzed	Not analyzed	1/15	1/50	1/60	1/30	1/30
**NeuN**	1/40	Not analyzed	Not analyzed	1/15	1/50	1/60	1/30	1/30
**DCX**	1/30	1/30	1/30	1/30	1/30	1/30	Not analyzed	Not analyzed
**Dissector Height (µm)**	**Birth**	**10 days**	**42 days**	**3 months**	**5 months**	**7 months**	**6 years**	**24 years**
	9			9	10	14	12	12
**Sampling Grid Size (µm)**	**Birth**	**10 days**	**42 days**	**3 months**	**5 months**	**7 months**	**6 years**	**24 years**
**DAPI Cortex**	842.8 x 927.5	Not analyzed	Not analyzed	1298.1 x 1045.	1740.6 x 1332.	1037.4 x 962	1346.5 x 1352.	1635.4 x 889.2
**NeuN Cortex**	573.4 x 631	Not analyzed	Not analyzed	894 x 719	1204 x 922	922 x 855	1206 x 1212	1417 x 770
**DCX**	500 x 500	400 x 400	500 x 500	450 x 450	500 x 500	500 x 500	Not Analyzed	Not Analyzed
**Coefficient of Error (Gundersen m=1)**	**Birth**	**10 days**	**42 days**	**3 months**	**5 months**	**7 months**	**6 years**	**24 years**
**DAPI Cortex**	0.05	Not analyzed	Not analyzed	0.05	0.05	0.05	0.05	0.05
**NeuN Cortex**	0.06	Not analyzed	Not analyzed	0.06	0.05	0.05	0.06	0.05
**DCX**	0.09	0.05	0.06	0.09	0.08	0.11	Not analyzed	Not analyzed

Table S4. Exhaustive Counts Parameters (Subtypes)

Section Sampling Fraction	1 day	3 months	5 months	7 months	6 years	24 years
**CalB**	1/30	1/15	1/40	1/40	1/70	1/20
**CalR**	1/20	1/2	1/40	1/30	1/20	1/20
**NPY**	1/25	1/15	1/50	1/60	1/40	1/20
**SST**	1/60	1/20	1/40	1/40	1/60	1/30
**PV**	N/A	1/3	1/30	1/50	1/35	1/25

Movie S1: 3D rendering of DCX+ cells (green) in Arc strip ensheathed by GFAP+ fibers (pink) at 1.5 months.

Movie S2: Time-lapse imaging showing a migrating neuron in a sagittal cortical slice from a brain at birth. Area imaged is developing white matter of cingulate. Note cell (*) is traveling in anterio-dorsal direction. Movie spans 18 hours.

Movie S3: Time-lapse imaging showing a migrating neuron in a coronal cortical slice from a brain at birth. Area imaged is at the dorsolateral edge of the lateral ventricle, within the Arc region. Movie shows a migrating neuron (*) leaving the dense cell collection. Movie spans 24 hours.

Movie S4: Time-lapse imaging showing migrating neuron in a coronal cortical slice from a brain at birth. Area imaged is at the dorsolateral edge of the lateral ventricle, within the Arc region. Movie shows migrating neuron (*) traveling alongside dense cell collection. Movie spans 24 hours.

Movie S5: Schematic animation of three-dimensional rendering of postnatally migrating neurons in the infant brain. Arc-orange, SVZ and RMS-magenta, MMS-green.

References

1. S. P. Gandhi, Y. Yanagawa, M. P. Stryker, Delayed plasticity of inhibitory neurons in developing visual cortex. *Proc. Natl. Acad. Sci. U.S.A.* **105**, 16797–16802 (2008). Medline doi:10.1073/pnas.0806159105

2. T. Toyoizumi, H. Miyamoto, Y. Yazaki-Sugiyama, N. Atapour, T. K. Hensch, K. D. Miller, A theory of the transition to critical period plasticity: Inhibition selectively suppresses spontaneous activity. *Neuron* **80**, 51–63 (2013). Medline doi:10.1016/j.neuron.2013.07.022

3. O. Marín, J. L. Rubenstein, A long, remarkable journey: Tangential migration in the telencephalon. *Nat. Rev. Neurosci.* **2**, 780–790 (2001). Medline doi:10.1038/35097509

4. S. Nery, G. Fishell, J. G. Corbin, The caudal ganglionic eminence is a source of distinct cortical and subcortical cell populations. *Nat. Neurosci.* **5**, 1279–1287 (2002). Medline doi:10.1038/nn971

5. T. Ma, C. Wang, L. Wang, X. Zhou, M. Tian, Q. Zhang, Y. Zhang, J. Li, Z. Liu, Y. Cai, F. Liu, Y. You, C. Chen, K. Campbell, H. Song, L. Ma, J. L. Rubenstein, Z. Yang, Subcortical origins of human and monkey neocortical interneurons. *Nat. Neurosci.* **16**, 1588–1597 (2013). Medline doi:10.1038/nn.3536

6. D. V. Hansen, J. H. Lui, P. Flandin, K. Yoshikawa, J. L. Rubenstein, A. Alvarez-Buylla, A. R. Kriegstein, Non-epithelial stem cells and cortical interneuron production in the human ganglionic eminences. *Nat. Neurosci.* **16**, 1576–1587 (2013). Medline doi:10.1038/nn.3541

7. A. A. Lavdas, M. Grigoriou, V. Pachnis, J. G. Parnavelas, The medial ganglionic eminence gives rise to a population of early neurons in the developing cerebral cortex. *J. Neurosci.* **19**, 7881–7888 (1999). Medline

8. H. Korr, C. Schmitz, Facts and fictions regarding post-natal neurogenesis in the developing human cerebral cortex. *J. Theor. Biol.* **200**, 291–297 (1999). Medline doi:10.1006/jtbi.1999.0992

9. R. L. Sidman, P. Rakic, Neuronal migration, with special reference to developing human brain: A review. *Brain Res.* **62**, 1–35 (1973). Medline doi:10.1016/0006-8993(73)90617-3

10. J. M. García-Verdugo, S. Ferrón, N. Flames, L. Collado, E. Desfilis, E. Font, The proliferative ventricular zone in adult vertebrates: A comparative study using reptiles, birds, and mammals. *Brain Res. Bull.* **57**, 765–775 (2002). Medline doi:10.1016/S0361-9230(01)00769-9

11. M. F. Paredes, S. F. Sorrells, J. M. Garcia-Verdugo, A. Alvarez-Buylla, Brain size and limits to adult neurogenesis. *J. Comp. Neurol.* **524**, 646–664 (2016). Medline doi:10.1002/cne.23896

12. C. Lois, J. M. García-Verdugo, A. Alvarez-Buylla, Chain migration of neuronal precursors. *Science* **271**, 978–981 (1996). Medline doi:10.1126/science.271.5251.978

13. R. Betarbet, T. Zigova, R. A. Bakay, M. B. Luskin, Dopaminergic and GABAergic interneurons of the olfactory bulb are derived from the neonatal subventricular zone. *Int. J. Dev. Neurosci.* **14**, 921–930 (1996). Medline doi:10.1016/S0736-5748(96)00066-4

14. P.-M. Lledo, A. Saghatelyan, M. Lemasson, Inhibitory interneurons in the olfactory bulb: From development to function. *Neuroscientist* **10**, 292–303 (2004). Medline doi:10.1177/1073858404263460

15. S. O. Suzuki, J. E. Goldman, Multiple cell populations in the early postnatal subventricular zone take distinct migratory pathways: A dynamic study of glial and neuronal progenitor migration. *J. Neurosci.* **23**, 4240–4250 (2003). Medline

16. D. Inta, J. Alfonso, J. von Engelhardt, M. M. Kreuzberg, A. H. Meyer, J. A. van Hooft, H. Monyer, Neurogenesis and widespread forebrain migration of distinct GABAergic neurons from the postnatal subventricular zone. *Proc. Natl. Acad. Sci. U.S.A.* **105**, 20994–20999 (2008). Medline doi:10.1073/pnas.0807059105

17. C. Le Magueresse, J. Alfonso, K. Khodosevich, A. A. Arroyo Martín, C. Bark, H. Monyer, "Small axonless neurons": Postnatally generated neocortical interneurons with delayed functional maturation. *J. Neurosci.* **31**, 16731–16747 (2011). Medline doi:10.1523/JNEUROSCI.4273-11.2011

18. S. De Marchis, A. Fasolo, A. C. Puche, Subventricular zone-derived neuronal progenitors migrate into the subcortical forebrain of postnatal mice. *J. Comp. Neurol.* **476**, 290–300 (2004). Medline doi:10.1002/cne.20217

19. C. Wang, F. Liu, Y. Y. Liu, C. H. Zhao, Y. You, L. Wang, J. Zhang, B. Wei, T. Ma, Q. Zhang, Y. Zhang, R. Chen, H. Song, Z. Yang, Identification and characterization of neuroblasts in the subventricular zone and rostral migratory stream of the adult human brain. *Cell Res.* **21**, 1534–1550 (2011). Medline doi:10.1038/cr.2011.83

20. N. Sanai, T. Nguyen, R. A. Ihrie, Z. Mirzadeh, H. H. Tsai, M. Wong, N. Gupta, M. S. Berger, E. Huang, J. M. Garcia-Verdugo, D. H. Rowitch, A. Alvarez-Buylla, Corridors of migrating neurons in the human brain and their decline during infancy. *Nature* **478**, 382–386 (2011). Medline doi:10.1038/nature10487

21. J. G. Gleeson, P. T. Lin, L. A. Flanagan, C. A. Walsh, Doublecortin is a microtubule-associated protein and is expressed widely by migrating neurons. *Neuron* **23**, 257–271 (1999). Medline doi:10.1016/S0896-6273(00)80778-3

22. K. Ono, H. Tomasiewicz, T. Magnuson, U. Rutishauser, N-CAM mutation inhibits tangential neuronal migration and is phenocopied by enzymatic removal of polysialic acid. *Neuron* **13**, 595–609 (1994). Medline doi:10.1016/0896-6273(94)90028-0

23. F. J. Martini, M. Valiente, G. López Bendito, G. Szabó, F. Moya, M. Valdeolmillos, O. Marín, Biased selection of leading process branches mediates chemotaxis during tangential neuronal migration. *Development* **136**, 41–50 (2009). Medline doi:10.1242/dev.025502

24. H. Wichterle, J. M. Garcia-Verdugo, D. G. Herrera, A. Alvarez-Buylla, Young neurons from medial ganglionic eminence disperse in adult and embryonic brain. *Nat. Neurosci.* **2**, 461–466 (1999). Medline doi:10.1038/8131

25. H. Wichterle, D. H. Turnbull, S. Nery, G. Fishell, A. Alvarez-Buylla, In utero fate mapping reveals distinct migratory pathways and fates of neurons born in the mammalian basal forebrain. *Development* **128**, 3759–3771 (2001). Medline

26. B. Nadarajah, J. E. Brunstrom, J. Grutzendler, R. O. Wong, A. L. Pearlman, Two modes of radial migration in early development of the cerebral cortex. *Nat. Neurosci.* **4**, 143–150 (2001). Medline doi:10.1038/83967

27. S. A. Anderson, O. Marín, C. Horn, K. Jennings, J. L. Rubenstein, Distinct cortical migrations from the medial and lateral ganglionic eminences. *Development* **128**, 353–363 (2001). Medline

28. E. Soriano, J. A. Del Rio, I. Ferrer, C. Auladell, L. De Lecea, S. Alcantara, Late appearance of parvalbumin-immunoreactive neurons in the rodent cerebral cortex does not follow an 'inside-out' sequence. *Neurosci. Lett.* **142**, 147–150 (1992). Medline doi:10.1016/0304-3940(92)90360-J

29. K. Letinic, I. Kostovic, Postnatal development of calcium-binding proteins calbindin and parvalbumin in human visual cortex. *Cereb. Cortex* **8**, 660–669 (1998). Medline doi:10.1093/cercor/8.7.660

30. O. Riccio, S. Murthy, G. Szabo, L. Vutskits, J. Z. Kiss, T. Vitalis, C. Lebrand, A. G. Dayer, New pool of cortical interneuron precursors in the early postnatal dorsal white matter. *Cereb. Cortex* **22**, 86–98 (2012). Medline doi:10.1093/cercor/bhr086

31. F. T. Merkle, Z. Mirzadeh, A. Alvarez-Buylla, Mosaic organization of neural stem cells in the adult brain. *Science* **317**, 381–384 (2007). Medline doi:10.1126/science.1144914

32. M. Sakamoto, N. Ieki, G. Miyoshi, D. Mochimaru, H. Miyachi, T. Imura, M. Yamaguchi, G. Fishell, K. Mori, R. Kageyama, I. Imayoshi, Continuous postnatal neurogenesis contributes to formation of the olfactory bulb neural circuits and flexible olfactory associative learning. *J. Neurosci.* **34**, 5788–5799 (2014). Medline doi:10.1523/JNEUROSCI.0674-14.2014

33. G. A. Cecchi, L. T. Petreanu, A. Alvarez-Buylla, M. O. Magnasco, Unsupervised learning and adaptation in a model of adult neurogenesis. *J. Comput. Neurosci.* **11**, 175–182 (2001). Medline doi:10.1023/A:1012849801892

34. A. Barnea, F. Nottebohm, Seasonal recruitment of hippocampal neurons in adult free-ranging black-capped chickadees. *Proc. Natl. Acad. Sci. U.S.A.* **91**, 11217–11221 (1994). Medline doi:10.1073/pnas.91.23.11217

35. L. Petreanu, A. Alvarez-Buylla, Maturation and death of adult-born olfactory bulb granule neurons: Role of olfaction. *J. Neurosci.* **22**, 6106–6113 (2002). Medline

36. E. Gould, A. Beylin, P. Tanapat, A. Reeves, T. J. Shors, Learning enhances adult neurogenesis in the hippocampal formation. *Nat. Neurosci.* **2**, 260–265 (1999). Medline doi:10.1038/6365

37. D. G. Southwell, R. C. Froemke, A. Alvarez-Buylla, M. P. Stryker, S. P. Gandhi, Cortical plasticity induced by inhibitory neuron transplantation. *Science* **327**, 1145–1148 (2010). Medline doi:10.1126/science.1183962

38. T. K. Hensch, Critical period plasticity in local cortical circuits. *Nat. Rev. Neurosci.* **6**, 877–888 (2005). Medline doi:10.1038/nrn1787

39. M. S. Lazarus, Z. J. Huang, Distinct maturation profiles of perisomatic and dendritic targeting GABAergic interneurons in the mouse primary visual cortex during the critical period of ocular dominance plasticity. *J. Neurophysiol.* **106**, 775–787 (2011). Medline doi:10.1152/jn.00729.2010

40. S. Robinson, Q. Li, A. Dechant, M. L. Cohen, Neonatal loss of gamma-aminobutyric acid pathway expression after human perinatal brain injury. *J. Neurosurg.* **104** (suppl.), 396–408 (2006). Medline

41. N. Salmaso, B. Jablonska, J. Scafidi, F. M. Vaccarino, V. Gallo, Neurobiology of premature brain injury. *Nat. Neurosci.* **17**, 341–346 (2014). Medline doi:10.1038/nn.3604

42. V. S. Catts, S. J. Fung, L. E. Long, D. Joshi, A. Vercammen, K. M. Allen, S. G. Fillman, D. A. Rothmond, D. Sinclair, Y. Tiwari, S. Y. Tsai, T. W. Weickert, C. Shannon Weickert, Rethinking schizophrenia in the context of normal neurodevelopment. *Front. Cell. Neurosci.* **7**, 60 (2013). Medline doi:10.3389/fncel.2013.00060

43. O. Marín, Interneuron dysfunction in psychiatric disorders. *Nat. Rev. Neurosci.* **13**, 107–120 (2012). Medline

44. R. Wright, V. Kyriakopoulou, C. Ledig, M. A. Rutherford, J. V. Hajnal, D. Rueckert, P. Aljabar, Automatic quantification of normal cortical folding patterns from fetal brain MRI. *Neuroimage* **91**, 21–32 (2014). Medline doi:10.1016/j.neuroimage.2014.01.034

45. H. Kim *et al*., in *Lecture Notes in Computer Science* (Springer, 2015), pp. 571–579.

46. L. Concha, H. Kim, A. Bernasconi, B. C. Bernhardt, N. Bernasconi, Spatial patterns of water diffusion along white matter tracts in temporal lobe epilepsy. *Neurology* **79**, 455–462 (2012). Medline doi:10.1212/WNL.0b013e31826170b6
